# Location and Species Matters: Variable Influence of the Environment on the Gene Flow of Imperiled, Native and Invasive Cottontails

**DOI:** 10.3389/fgene.2021.708871

**Published:** 2021-09-29

**Authors:** Thomas J. McGreevy, Sozos Michaelides, Mihajla Djan, Mary Sullivan, Diana M. Beltrán, Bill Buffum, Thomas Husband

**Affiliations:** ^1^ Department of Natural Resources Science, University of Rhode Island, Kingston, RI, United States; ^2^ Department of Biology, Concordia University, Montreal, QC, Canada; ^3^ Department of Biology and Ecology, Faculty of Sciences, University of Novi Sad, Novi Sad, Serbia; ^4^ USDA Agricultural Research Service, National Cold Water Marine Aquaculture Center, Kingston, RI, United States

**Keywords:** ddRADSeq, eastern cottontail (*Sylvilagus floridanus*), geographic information systems, landscape genetics, New England cottontail (*Sylvilagus transitionalis*), random forest, specialist-generalist variation hypothesis

## Abstract

The environment plays an important role in the movement of individuals and their associated genes among populations, which facilitates gene flow. Gene flow can help maintain the genetic diversity both within and between populations and counter the negative impact of genetic drift, which can decrease the fitness of individuals. Sympatric species can have different habitat preferences, and thus can exhibit different patterns of genetic variability and population structure. The specialist-generalist variation hypothesis (SGVH) predicts that specialists will have lower genetic diversity, lower effective population sizes (Ne), and less gene flow among populations. In this study, we used spatially explicit, individual-based comparative approaches to test SGVH predictions in two sympatric cottontail species and identify environmental variables that influence their gene flow. New England cottontail (*Sylvilagus transitionalis*) is the only native cottontail in the Northeast US, an early successional habitat specialist, and a species of conservation concern. Eastern cottontail (*S. floridanus*) is an invasive species in the Northeast US and a habitat generalist. We characterized each species’ genomic variation by developing double-digest Restriction-site Associated DNA sequence single nucleotide polymorphism markers, quantified their habitat with Geographic Information System environmental variables, and conducted our analyses at multiple scales. Surprisingly, both species had similar levels of genetic diversity and eastern cottontail’s Ne was only higher than New England cottontail in one of three subregions. At a regional level, the population clusters of New England cottontail were more distinct than eastern cottontail, but the subregional levels showed more geographic areas of restricted gene flow for eastern cottontail than New England cottontail. In general, the environmental variables had the predicted effect on each species’ gene flow. However, the most important environmental variable varied by subregion and species, which shows that location and species matter. Our results provide partial support for the SGVH and the identification of environmental variables that facilitate or impede gene flow can be used to help inform management decisions to conserve New England cottontail.

## Introduction

The environment has an important effect on the movement of individuals and their associated genes among populations. Gene flow can help maintain the genetic diversity both within and between populations and counter the negative impact of genetic drift. When populations become isolated, random alleles can become fixed. This results in the loss of genetic diversity and an increase in inbreeding, which both have negative impacts on the fitness of individuals (Frankham et al., 2017). Genetic factors (e.g., genetic diversity and inbreeding level) play a central role in the response to the challenges confronting wildlife (Hohenlohe et al., 2020). Studies focused on associations between relevant environment variables and fine-scale genetic data shed light on key elements affecting population fitness and provide informed-based conservation decisions (Frankham et al., 2017). Understanding the effects of environmental factors on genetic variability and structure also is crucial for long-term ecological management. An important first step is to characterize environmental factors in specific landscapes, quantify how those factors affect genetic variability, and to study contemporary gene flow to determine the potential for adaptive genes to spread (Manel and Holderegger, 2013). However, the environmental variables selected for analyses should be appropriate for the specific study and reasonably selected based on the ecology of the species and knowledge regarding the habitat used (Storfer et al., 2018). The identification of key environmental variables that shape genetic variability and influence gene flow at an intraspecific level provide scientific-based information to conservation management and point to priority targets for the long-term sustainability of species. Combining this information can provide a prediction of future patterns of spatial genetic variation under different environmental scenarios.

Closely related species with overlapping ranges can have different habitat preferences, and thus can exhibit different patterns of genetic variability and population structure (Kraaijeveld-Smit et al., 2007). In Leporids, this was the case for habitat specialist swamp rabbit (*Sylvilagus aquaticus*) and habitat generalist eastern cottontail (*S. floridanus*) in Southern Illinois (Berkman et al., 2015), where profound population structuring was found for swamp rabbits, accompanied by lower genetic variability and effective population size (Ne). No such pattern was observed for eastern cottontails. The main environmental factors responsible for the observed patterns were habitat fragmentation and restricted dispersal corridors, as well as the linear nature of swamp rabbit habitat. The results of Berkman et al. (2015) have been used by Matthee et al. (2018) as a terrestrial vertebrate example that supports the specialist-generalist variation hypothesis (SGVH; Li et al., 2014). The SGVH predicts that host specialists will have lower Ne and less gene flow among populations than host generalists (Li et al., 2014). The SGVH was developed for invertebrates and support for the hypothesis has been found with nematodes (Li et al., 2014), parasitic mites (Matthee et al., 2018), and ectoparasites (Wessels et al., 2019). However, the SGVH has yet to be explicitly applied to vertebrates to test its predictive generality and see if it extends beyond parasite-host relationships.

The evolution of a specialist-generalist strategy can be a highly dynamic process during the evolutionary history of naturally co-occurring species and may play an important role in species diversification (Li et al., 2014) and population genetic structure. When a native species exhibits narrow habitat preference and fragmented populations in a patchy environment, it also is expected to be highly influenced by genetic drift and inbreeding, and subsequently have decreased genetic variability and evolutionary potential (Hedrick, 2011). The establishment of a closely-related invasive species that can use a wider variety of habitats in the same region may cause even more profound effects on the native species.

In our study system, we focus on the comparative analyses of sympatric cottontails in the Northeast United States. New England cottontail (*S. transitionalis*) is the only native cottontail in the Northeast US and is listed as vulnerable by the IUCN Redlist (Litvaitis and Lanier, 2019). New England cottontail is an early successional habitat specialist (Litvaitis, 1993), which has had a declining distribution over the past 2 decades (Litvaitis et al., 2006; Rittenhouse and Kovach, 2021). Their decline has been attributed to habitat loss, habitat fragmentation, and competition with the nonnative eastern cottontail (Litvaitis et al., 2008). Eastern cottontails are nonnative in the Northeastern US and were introduced east of the Hudson River starting in the early 1900s. State biologists and hunting clubs translocated hundreds of thousands of eastern cottontails that included at least five subspecies (Johnston, 1972; Litvaitis et al., 2008). Eastern cottontail include over 30 subspecies (Nielsen and Berkman 2018) and the intermixing of numerous eastern cottontail subspecies has undoubtedly led to the increase in their genetic diversity. Eastern cottontail in the Northeast have retained nearly nine times the number of mitochondrial DNA haplotypes compared to New England cottontail (Litvaitis et al., 1997; Sullivan et al., 2019). The higher level of variation in genotype and phenotype of introduced eastern cottontail also has been documented in Maryland where over 200,000 eastern cottontail were translocated to the state (Chapman and Morgan 1973). Eastern cottontail is a habitat generalist that can be found in a wide variety of habitats that range from rural to urban (Hunt et al., 2014) and span from North to South America (Chapman et al., 1980). The introduction of eastern cottontail may represent one of the causes that accelerated the decrease in number of New England cottontail and loss of their habitat.

Previous research on the population genetics of New England cottontail, based on a limited number of microsatellite markers, has identified four genetically disjunct populations that have differentiated due to drift (Fenderson et al., 2011). Microsatellite marker analyses also have been conducted in eastern New York on New England cottontail and found that the species is disjunct with minimal gene flow among sites within that population (Cheeseman et al., 2019). More recent population genomic analyses, based on the analyses of thousands of single nucleotide polymorphism (SNP) markers using various methods, has confirmed these same four geographic populations for New England cottontail and found a similar general pattern for eastern cottontail (Neil 2020). At the regional level, eastern cottontail show a more gradual transition among populations and population level analyses show no evidence for isolation by distance, while New England cottontail do show isolation by distance (Neil 2020; Wright, 1943). Landscape genetic analyses on New England cottontail are limited and based on the analyses of a limited number of microsatellite markers. In New England cottontail’s uppermost Northeast range area, landscape genetic analyses have identified anthropogenic roadside shrublands and natural shrublands as facilitating gene flow and roads, development, and forest as impeding gene flow (Fenderson et al., 2014; Amaral et al., 2016).

Increasingly, it is recognized that comparative analyses and replication are needed to make more robust conclusions and advance the field of landscape genetics (Richardson et al., 2016; Waits et al., 2016; Storfer et al., 2018). Comparative landscape genetic analyses on nuclear DNA have yet to be conducted on both species and can help inform management decisions to conserve New England cottontail. The objectives of our study were to: 1) characterize the neutral genetic diversity of New England cottontail and eastern cottontail; 2) identify environmental variables that influence each species’ gene flow; and 3) test the SGVH. We characterized genomic variation by using a reduced representation approach to develop double-digest Restriction-site Associated DNA sequence (dd-RADseq; Peterson et al., 2012) SNP markers. We hypothesize that New England cottontail will have lower genetic diversity and Ne than eastern cottontail because New England cottontail are only found in remnant, disjunct populations that are in decline. We also hypothesize that New England cottontail’s gene flow will be more restricted than eastern cottontail’s gene flow and environmental variables will explain less of the genetic differentiation among eastern cottontails (i.e., stronger barriers to New England cottontail’s gene flow). To address these objectives we used a spatially explicit, individual based approach to determine if environmental variables have shaped New England and eastern cottontail population structure, identified geographic areas of restricted gene flow, and quantified the impact of environmental variables on their genetic differentiation. We conducted our analyses at both regional and subregional scales, and selected subregions where both species are sympatric and considered one population. The subregions, in general, matched the subregions defined by Neil (2020) and were delineated to maximize the number of samples included for both species within an area. The analyses within a population were conducted to reduce the confounding effect of population structure and provide replicate sites (Chikhi et al., 2010; Richardson et al., 2016). We selected environmental variables that previously have been identified as influencing New England cottontail gene flow. Our results provide partial support for the SGVH and the identification of environmental variables that facilitate and impede gene flow can be used to help inform management decisions to conserve New England cottontail.

## Materials and Methods

### Sample Collection and DNA Extraction

**FIGURE 1:**
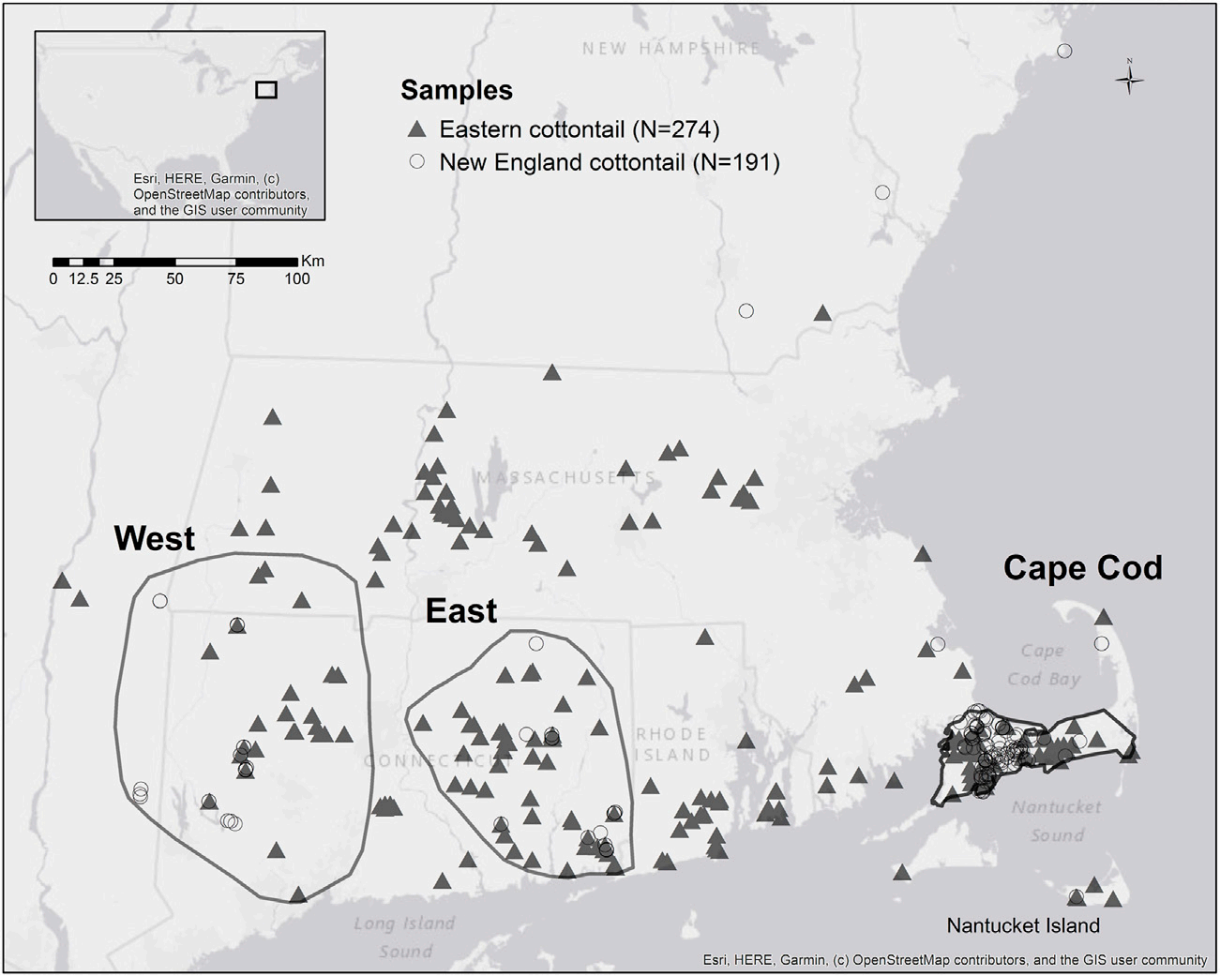
Map of New England cottontail (*Sylvilagus transitionalis*) and eastern cottontail (*S. floridanus*) samples with the polygons showing the subregions West, East, and Cape Cod.

We received 191 and 274 tissue samples from New England (NEC) and eastern cottontail (EAC), respectively, collected by zoo personnel, state biologists, and federal biologists. The samples were collected from 2010 to 2016 from the New England area and eastern New York. The full range wide samples for each species were subdivided into three subregions: Cape Cod (NEC *n* = 114; EAC *n* = 70), West (NEC *n* = 31; EAC *n* = 29), and East (NEC *n* = 31; EAC *n* = 59). The subregions were selected to maximize the number of samples from each species, while still remaining in the general area of a single population for both species (Figure 1). The tissue samples were stored in >95% ethanol and transported at room temperature to the Wildlife Genetics and Ecology Laboratory at the University of Rhode Island where they were stored at −20°C prior to DNA extraction. Genomic DNA was extracted from the tissue samples using either the DNeasy Blood and Tissue kit (Qiagen Sciences, MD) or the NucleoMag Tissue kit (Macherey-Nagel Inc., PA) according to manufacturers’ instructions. All sets of DNA extractions included a negative control to test for contamination in the reagents.

### ddRADseq Library Preparation and Sequencing

We followed the Peterson et al. (2012) approach for producing the double digest Restriction-site Associated DNA sequencing (ddRADseq) libraries. Briefly, extracted DNA was quantified using the high-sensitivity Quant-iT^TM^ dsDNA Assay Kit (Invitrogen) on a BioTek Synergy^TM^ H1 (BioTek Instruments, Inc., VT) microplate reader. The initial quantity of DNA was standardized to 400 ng for each sample using an epMotion 5075 TMX (Eppendorf, CT) and digested using the restriction enzymes SphI-HF (New England Biolabs, Ipswich, MA) and MluCI (New England Biolabs, Ipswich, MA). Digested DNA was purified using NucleoMag 96 PCR cleanup kit (Macherey-Nagel Inc., PA), quantified, and standardized to 150–250 ng. Sets of 12 samples were barcoded with unique P1/P2 adapters, purified, and combined at equal concentrations. The adapter labelled pools were size selected for 333 bp to 414 bp using ethidium free gels on the Pippin Blue (Sage Scientific, MA) system and cleaned using Dynabeads (Thermofisher Scientific, MA) to remove fragments of DNA that did not have a forward and reverse adapter attached. Four sets of pooled samples containing 48 samples were polymerase chain reaction (PCR) amplified for 12–15 cycles using the Kapa Library Amplification Kit (Kapa Biosystems, MA) according to manufacturer’s instructions to add Illumina (San Diego, CA) flowcell annealing sequences, multiplexing indices, and to increase the amount of labelled fragments. Index labelled pools were purified and quantified using the Agilent High Sensitivity DNA kit (Agilent Technologies, Santa Clara, CA) and the Kapa Library Quantification Kit (Kapa Biosystems, Wilmington, MA) on a LightCycler® 480 (Roche, Switzerland) according to manufacturers’ instructions. The index labelled pools were combined at equal concentrations, purified, and quantified again using the Agilent High Sensitivity DNA kit. The final pooled library was submitted to the Northwest Laboratory Bauer Core Facility at Harvard University for single end 50 bp read sequencing using an Illumina HiSeq™ 2000 (San Diego, CA). A total of six libraries were sequenced.

### Single Nucleotide Polymorphism Data Quality Control Filtering and Calling

The Bauer Core Facility staff demultiplexed the raw next-generation sequencing data by each index. The University of Rhode Island’s Bluewaves high performance computing and R version 3.6.2 (R Core Team 2019) through RStudio version 1.2.5033 (RStudio Team 2019) were used to run all software programs for SNP quality control filtering and calling. The demultiplexed reads were processed separately for each species using STACKS v.2.4 to v.2.53 (Catchen et al., 2011; Catchen et al., 2013; Rochette et al., 2019). The *process_radtag* program was used to conduct the initial quality control filtering and assign reads to individuals with the settings -c, -q, and -r. We qualitatively assessed the sequence read quality using the software FastQC (http://www.bioinformatics.babraham.ac.uk/projects/fastqc/). The cleaned reads were aligned to whole genome sequences generated from New England cottontail DNA using the program BWA v.0.7.17 (Li and Durbin, 2009). The whole genome sequences were produced by the United States Geological Survey using the Ion Torrent PGM and Ion Proton platforms (Gilford, NH), which produced 1.75 million contigs that averaged 1,680 base pairs. The contigs were mapped to the *Oryctolagus cuniculus* genome using CLC Bio (Aarhus, Denmark), which retained 89% of the contigs. The *ref_map.pl* pipeline in STACKS was used to process the cleaned reads that were aligned to the New England cottontail contigs. The *populations* program in STACKS was used to call SNPs using the parameter settings -R 0.8, --min-maf 0.01, --min-mac 3, and --max-obs-het 0.7. The retained SNPs were further filtered using the programs vcftools v.1.16 (Danecek et al., 2011) and *mawk* (https://invisible-island.net/mawk/) was used to filter samples that had more than 50% missing data. Potential adaptive SNPs were filtered using the program *PCAdapt* (Luu et al., 2016) with an alpha value of 0.1. Lastly, SNPs that did not conform to Hardy-Weinberg equilibrium (HWE) were filtered using the program *filter_hwe_by_pop.pl* (https://github.com/jpuritz/dDocent/blob/master/tutorials/Filtering%20Tutorial.md) with a minimum *p*-value cutoff of 0.01 and all samples considered one population. Replicate samples were processed for New England cottontail (*n* = 47) and eastern cottontail (*n* = 23) within each library run. The program *SNPRelate* (Zheng et al., 2012) was used to confirm replicate samples were nearly identical. Replicate samples that passed quality control filtering were merged for New England cottontail (*n* = 39) and eastern cottontail (*n* = 23).

### Genetic Diversity and Population Structure

Average observed heterozygosity (Ho), expected heterozygosity (He), and inbreeding (F_IS_) was estimated at the regional and subregional scales using the program *populations* in STACKS. The R package *sGD* (Shirk and Cushman, 2011) was used to estimate spatially explicit Ho for each species at an individual level. A neighborhood distance of 40 km was used at the regional scale and a distance of 10 km was used at the subregional scale to maximize the number of samples included in the analyses. Effective population sizes (Ne) were estimated for each species at the subregional scale (Cape Cod, West, and East) using the linkage disequilibrium model based on single moment data as implemented in NeESTIMATOR v2 (Do et al., 2014). Mean Ne estimates were made using allele frequencies >0.01, and 95% confidence intervals were established from jackknife cross-validations.

The presence of isolation by distance can confound population structure analyses (Perez et al., 2018). We tested for isolation by distance at the individual level by comparing the genetic difference matrices to geographic distance using GenAlEx 6.502 (Peakall and Smouse 2006; Peakall and Smouse 2012) with 9,999 permutations and an alpha value of 0.05. Population structure was characterized using the R package *adegenet* (Jombart, 2008) to conduct Discriminate Analysis of Principal Components (DAPC) (Jombart et al., 2010). We used the general approach described in the DAPC tutorial by Jombart and Collins (2015) to conduct the analyses. We used two spatially explicit analyses, Estimate of Effective Migration Surfaces (Petkova et al., 2016; EEMS) and MEMGENE (Galpern et al., 2014), to identify areas of restricted gene flow and determine if environmental variables may be influencing gene flow. The program EEMS is based on a stepping stone model (Kimura and Weiss, 1964) and identifies geographic areas that depart from an isolation by distance model. For the EEMS analyses we used the script *bed2diffs_v2* to estimate the difference matrix, which imputes missing SNP data and calculates the average pairwise difference among all individuals. The proposal variances were optimized between 20 and 30% as described by the EEMS instruction manual (https://github.com/dipetkov/eems/blob/master/Documentation/EEMS-doc.pdf) using a deme size of 100. We conducted three independent runs with different deme sizes (400, 800, and 1,000), averaged the runs, and displayed the results using R scripts described in the EEMS instruction manual (https://github.com/dipetkov/eems/blob/master/Documentation/EEMS-doc.pdf). The MEMGENE analyses were conducted on the proportion of shared alleles (Dps). We used the R package *propShared* in *adegenet* to estimate Dps, which is a measure of gene flow. The MEMGENE analyses identifies independent spatial axes of genetic variation and estimates the percent of genetic variance explained by each axis. This analysis determines if environmental variables are potentially influencing gene flow and quantifies the amount of genetic variation explained by a given spatial pattern. The MEMGENE analyses were conducted at the regional and subregional scale.

### Environmental Variation Analyses

The individual Dps values were analyzed using the R package *randomForest* (Liaw and Wiener, 2002). The Cape Cod subregion was reduced to remove the northernmost samples at the National Sea Shore to reduce the inclusion of missing environmental data due to the ocean (Figure 1), which decreased the New England cottontail sample size to 107 and the eastern cottontail sample size to 67. The Random Forest method (Breiman, 2001) is a regression-based machine learning algorithm that was first applied to a landscape genetic analysis by Murphy et al. (2010) to identify environmental variables that influence genetic connectivity. The ability of the Random Forest approach to identify relevant environmental variables that influence genetic differentiation was tested with empirical data and simulations by Heather and Hoffman (2012) and found to be robust. The R script for running the *randomForest* analyses was developed by the Yale Landscape Genetics workshop. The parameters for the *randomForest* analyses were tuned using the settings stepFactor = 1.5, improve = 1e^−5^, and ntree = 500. The ntree parameter was increased until the percent variation explained peaked. The analyses were run three times for each species and subregion to test for the consistency of the results, because each run randomly subsets a portion of the data for validation. The straight linear distance between sample points was used to estimate the average values of each environmental variable. The seven environmental variables were all at a 30 m pixel resolution and were obtained from freely available database websites. The variables included elevation based on a digital elevation model (DEM; landfire.gov), aspect (landfire.gov), forest height which is a LiDAR derived product (Potapov et al., 2020), distance to medium and high development (landfire.gov), shrub vegetation cover (landfire.gov), forest and herbaceous wetlands (National Land Cover Data, USGS.gov), and roads (United States Major Roads from ESRI). The expected impact of each environmental variable on the genetic differentiation among individuals of each species is described in Table 1. The environmental variables were initially processed in ArcMap 10.5.1 (ESRI, CA) to standardize their projection and coordinate system and estimate the distances to the relative environmental variable. The extent of each raster environmental variable layer was standardized in R. The importance of each environmental variable was quantified using the *importance* function in the *randomForest* package, which estimates the percent increase in mean square error (MSE) and increase in node purity. The percent increase in MSE is estimated by first permuting the out of bag (OOB) data, which are the data removed for testing the prediction accuracy, and estimating the MSE. Each predictor variable is then permuted and the difference between the permuted variable and OOB data is averaged across all trees and normalized by their standard deviation. The increase in node purity is estimated using residual sum of squares and is the impact of splitting on a given predictor variable, which is averaged over all trees (https://cran.r-project.org/web/packages/randomForest/randomForest.pdf). The influence of the top importance variables was visualized by creating partial dependence plots in R using the *partialPlot* function.

**TABLE 1:** Environmental variables and their predicted influence on New England cottontail (*Sylvilagus transitionalis*; NEC) and eastern cottontail (*S. floridanus*; EAC) gene flow.

Variable	Description	Genetic prediction	Comparative prediction	References
Aspect	Slope direction	Neutral influence on gene flow	NEC = EAC	Desta et al. (2004)
Elevation	Elevation above sea level	Neutral influence on gene flow	NEC = EAC	—
Dist_dev	Distance to medium and high development	Lower values reduce gene flow	NEC > EAC	Amaral et al. (2016)
Dist_road	Distance to nearest road	Lower values will reduce gene flow	NEC > EAC	Fenderson et al. (2014), Amaral et al., 2016
Dist_shrub	Distance to shrub vegetation cover	Lower values will increase gene flow	NEC > EAC	Fenderson et al. (2014), Amaral et al. (2016)
Dist_wetland	Distance to forest and herbaceous wetlands	Lower values will increase gene flow	NEC > EAC	Amaral et al. (2016), Buffum et al. (2015)
Forest_height	Forest canopy height	Higher values decrease gene flow	EAC > NEC	Amaral et al. (2016), Buffum et al. (2015), Cheeseman et al. (2018), Mayer et al. (2018)

## Results

### Single Nucleotide Polymorphism Data Quality Control Filtering and Calling

The six library runs produced 807,714,829 million reads after filtering with *process_radtags*. The average number of reads per individual New England cottontail was 1,695,987 +/− 907,637 (standard deviation) and per individual eastern cottontail was 1,425,021 +/− 1,549,282. The PCAdapt and HWE outlier filtering removed 113 (57 PCAdapt + 56 HWE) and 429 (41 PCAdapt + 388 HWE) SNPs from the New England cottontail and eastern cottontail data sets, respectively. The number of retained SNPs ranged from 1,725 to 3,318 for New England cottontail and 4,085 to 14,029 for eastern cottontail. The cleaned fastq files are deposited in NCBI’s SRA database under BioProject accession number PRJNA736852.

### Genetic Diversity, Population Structure, and Gene Flow

**FIGURE 2:**
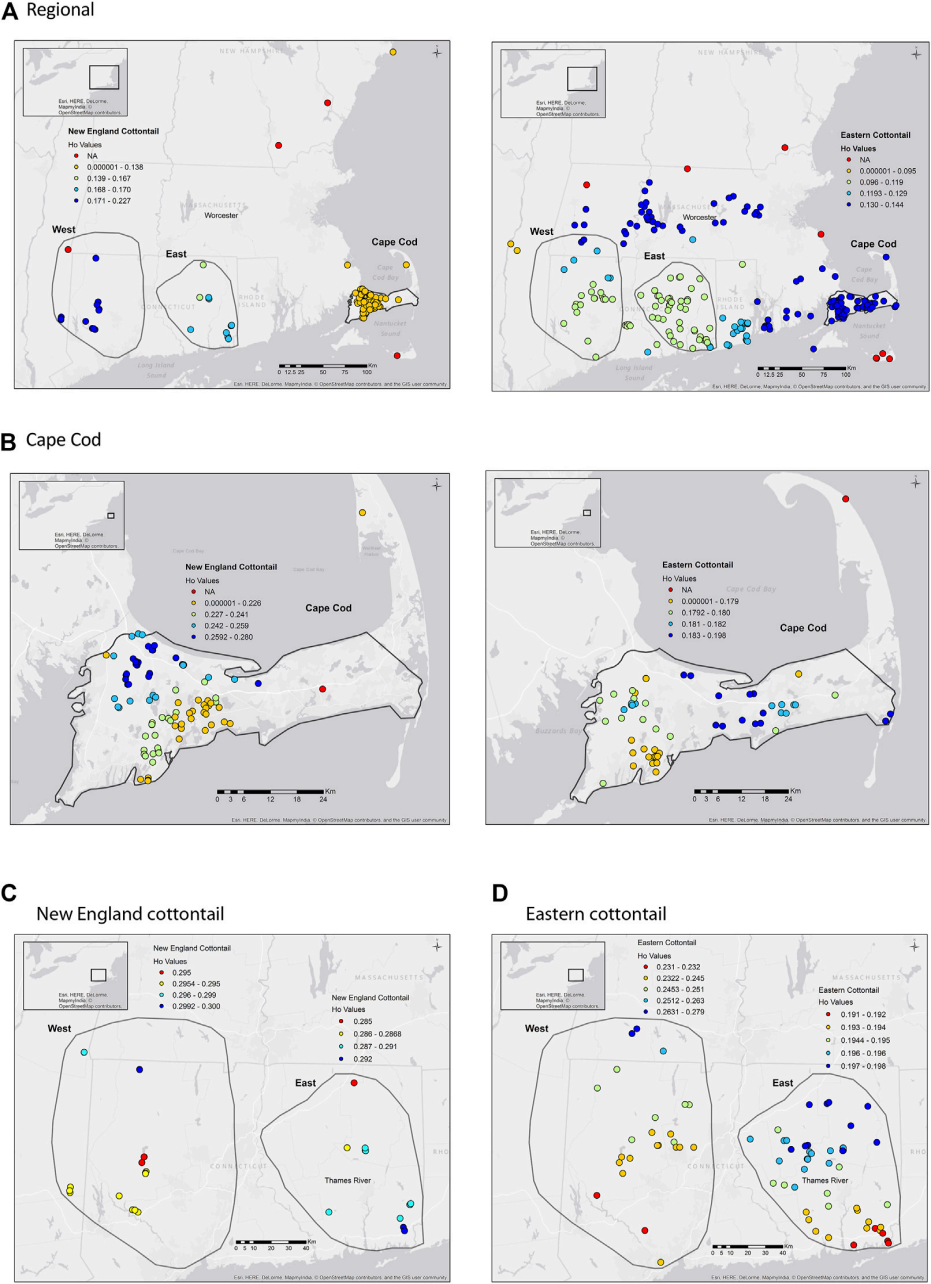
*sGD* (Shirk and Cushman 2011) observed heterozygosity (Ho) for New England cottontail (*Sylvilagus transitionalis*; NEC) and eastern cottontail (*S. floridanus*; EAC) at regional and subregional scales. The colors represent the Ho value, while the red dots are sample locations that did not have enough samples to estimate Ho for Figures A and B. **(A)** NEC (Region) EAC (Region). **(B)** NEC (Cape Cod) EAC (Cape Cod). **(C)** NEC (West) NEC (East). **(D)** EAC (West) EAC (East).

The average Ho and He values were higher for New England cottontail compared to eastern cottontail values at the regional and subregional scales (Table 2). The highest Ho and He values for both species were in the West subregion, which was the largest geographic extent. The inbreeding value was the highest for New England cottontail at the regional scale. However, in the Cape Cod subregion the eastern cottontail F_IS_ value was over three times higher than New England cottontail. Estimates of Ne for each species at the subregional scale ranged from 14.9 (NEC East) to 264.1 (EAC East) with New England cottontail having lower overall values across subregions. However, eastern cottontail only had significantly higher Ne (nonoverlapping confidence intervals) than New England cottontail in the East subregion. For New England cottontail the highest Ne estimate was in the Cape Cod subregion. The sGD estimate of individual Ho ranged from 0.125 to 0.300 for New England cottontail and 0.100 to 0.270 for eastern cottontail, but the values changed depending upon the scale of analysis (Figure 2). At a regional scale for New England cottontail, the Ho values increased from east to west, but the Ho values for eastern cottontail increased from southwest to northeast. For both species, the highest sGD estimates of Ho were in the West subregion. Both species showed an increase in Ho values from south to north in the West subregion. In the East subregion, eastern cottontail showed the same transition from low to high Ho values, but New England cottontail showed higher Ho values in the south. The Ho values for New England cottontail on Cape Cod were the highest in the northwest corner, while the values increased towards the east for eastern cottontail.

**TABLE 2:** Sample size (n), number of single nucleotide polymorphisms (SNPs) and average observed heterozygosity (Ho), expected heterozygosity (He), and inbreeding (F_IS_) estimated at the regional and subregional scales for New England cottontail (*Sylvilagus transitionalis*; NEC) and eastern cottontail (*S. floridanus*; EAC) using the program *populations* in STACKS (Catchen et al., 2011; Catchen et al., 2013). Variation in each average is represented by the standard error (SE). Effective population size (Ne) and 95% confidence intervals (CI) using the linkage disequilibrium method were estimated for each subregion using NeEstimator (Do et al., 2014).

Population	n	SNPs	Ho (SE)	He (SE)	F_IS_ (SE)	Ne (95%CI)
NEC Regional	191	3,318	0.153 (0.003)	0.179 (0.003)	0.168 (0.174)	—
NEC Cape Cod	120	1,725	0.242 (0.004)	0.249 (0.004)	0.030 (0.141)	93.2 (53.5–213.1)
NEC West	31	2,332	0.296 (0.003)	0.308 (0.003)	0.055 (0.030)	43.1 (25.7–98.3)
NEC East	31	1,810	0.292 (0.004)	0.300 (0.003)	0.042 (0.034)	14.9 (7.6–34.2)
EAC Regional	274	14,029	0.125 (0.001)	0.146 (0.001)	0.128 (0.129)	—
EAC Cape Cod	70	10,010	0.183 (0.001)	0.204 (0.001)	0.110 (0.035)	134.1 (96.6–222.1)
EAC West	29	4,085	0.249 (0.002)	0.259 (0.002)	0.058 (0.021)	67.4 (34.7–331.6)
EAC East	59	7,348	0.195 (0.002)	0.203 (0.002)	0.054 (0.035)	264.1 (141.3–1,312.7)

The regional analyses of isolation by distance was significant for both species with an R^2^ value more than five times higher for New England cottontail than eastern cottontail (Supplementary Appendix S1). At the subregional level for New England cottontail, the West (R^2^ = 0.139; *p* = 0) and East (R^2^ = 0.185; *p* = 0) regions showed a significant isolation by distance pattern, while the Cape Cod analysis was not significant (R^2^ = 0.002; *p* = 0.241). All three subregional analyses for eastern cottontail were significant: West (R^2^ = 0.148; *p* = 0.010), East (R^2^ = 0.016; *p* = 0.032) and Cape Cod (R^2^ = 0.118; *p* = 0).

The regional DAPC analyses showed the expected clustering of New England cottontail samples into distinct West, East, and Cape Cod clusters (Supplementary Appendix S2). The eastern cottontail samples showed a more gradual transition among the subregions and interestingly, a separate cluster in the western section of Cape Cod. The samples on Nantucket Island from both species clustered with samples from the East subregion. At the subregional level, the DAPC analyses showed the separation of two clusters for three of the six analyses (Supplementary Appendix S2). In the East subregion, both species showed a separation of samples from the southeast area and eastern cottontail showed a separation in the west on Cape Cod. Each analysis showed two clusters with varying amounts of overlap in the distributions of the discriminant functions. In general, the subregions that had the most clear separation of clusters also had the tightest peaks and least amount of overlap in their discriminant function peaks.

**FIGURE 3:**
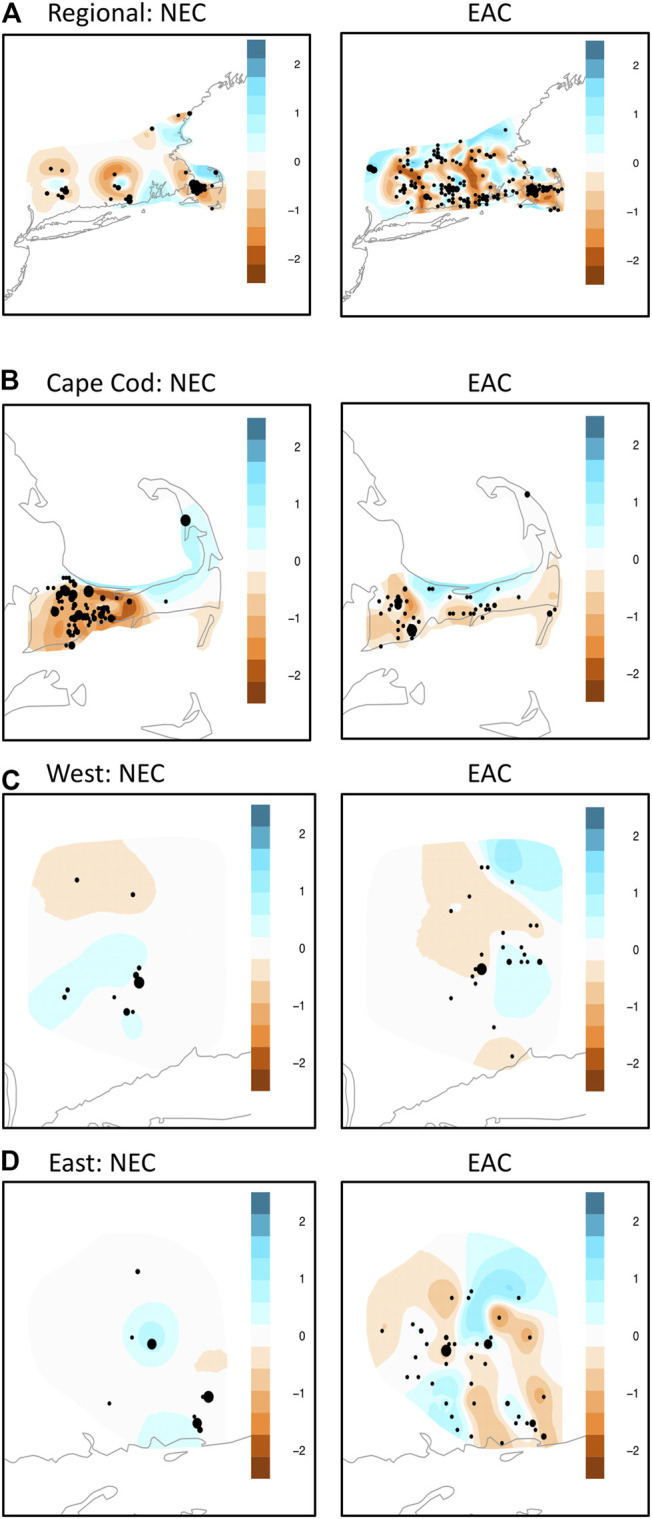
Estimate of Effective Migration Surfaces (Petkova et al., 2016) analyses of New England cottontail (*Sylvilagus transitionalis*; NEC) and eastern cottontail (*S. floridanus*; EAC) at the regional and subregional scale. Colors represent averaged posterior mean migration rates on the log10 scale. The different sized dots represent how many samples were aggregated together in a given location. **(A)** NEC (Region) EAC (Region). **(B)** NEC (Cape Cod) EAC (Cape Cod). **(C)** NEC (West) EAC (West). **(D)** NEC (East) EAC (East).

Overall, the EEMs results showed a more complex pattern of gene flow for eastern cottontail compared to New England cottontail due to more areas of facilitated and restricted gene flow (Figure 3). At the regional level, both species showed the same general pattern of restricted areas of gene flow in Cape Cod, East, and West subregion areas. The EEMs results for Cape Cod showed a similar pattern of barriers to gene flow in the west for each species with the barrier stronger for New England cottontail compared to eastern cottontail. Both species also showed a large area of higher than expected gene flow in the upper part of Cape Cod. However, eastern cottontail showed multiple areas of restricted gene flow rather than just one large area. The West subregion also showed a somewhat similar pattern between the two species with an area of restricted gene flow in the northwest and facilitated gene flow just south. However, eastern cottontail showed an additional area of restricted gene flow in the southernmost area and facilitated gene flow in the northeastern area. Eastern cottontail showed a more varied pattern in the East region with multiple areas of restricted and facilitated gene flow, whereas New England cottontail only showed one relatively small area of restricted and two areas of facilitated gene flow.

The adjusted R-squared values, amount of genetic variation explained by a given spatial pattern, for the New England cottontail MEMGENE analyses ranged from a negative value to 0.13. The highest amount of genetic variation was explained at the regional scale, whereas Cape Cod and the West subregions essentially had zero. The first eigenvalue axis for the regional and East subregion explained the majority of genetic variation at 55 and 69%, respectively. The adjusted R-squared values for eastern cottontail MEMGENE analyses ranged from 0.01 to 0.11, with the regional scale explaining the highest amount of genetic variation and the West subregional scale the lowest. The first eigenvalue axis for the regional, Cape Cod, and West subregions explained the majority of genetic variation at 44, 54, and 35%, respectively. The percent of variation explained by the East subregion’s eigenvalues was more evenly distributed among numerous axes and ranged from 10 to 17%.

**FIGURE 4:**
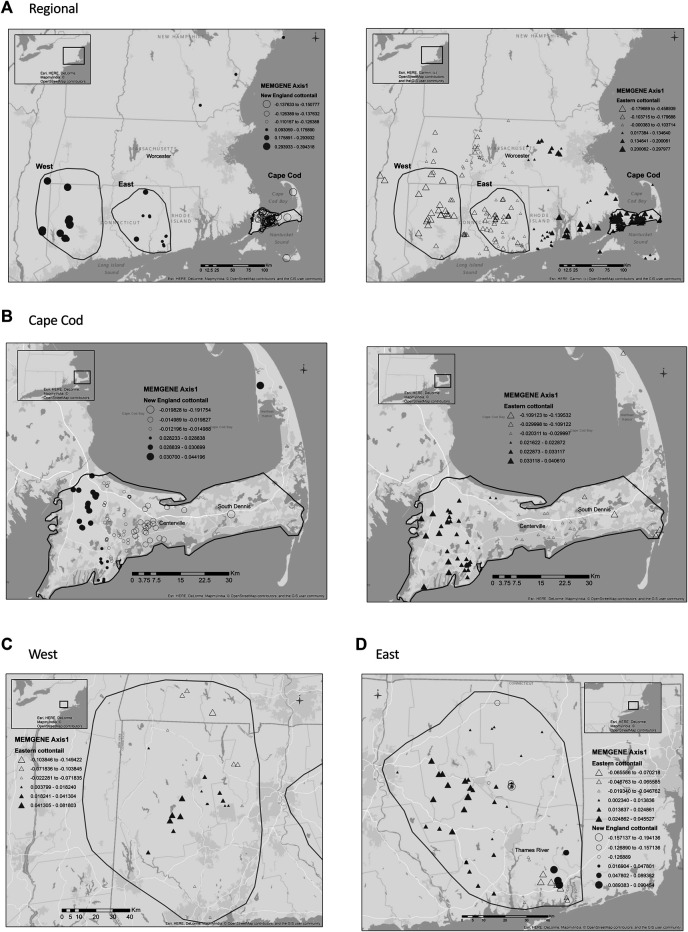
MEMGENE (Galpern et al., 2014) analyses of New England cottontail (*Sylvilagus transitionalis*; NEC) and eastern cottontail (*S. floridanus*; EAC) at regional and subregional scales. For each species, the positive and negative eigenvector values for the first memegene axis are separated. The size of the symbol relates to the given value with values closest to 0 being the smallest.**(A)** Regional. **(B)** Cape Cod. **(C)** West. **(D)** East.

The regional map of New England cottontail’s first MEMGENE axis showed four groups that generally corresponded with samples from New Hampshire/Maine, Cape Cod, eastern Connecticut, and western Connecticut/New York (Figure 4). The strongest separation occurred between samples from eastern Massachusetts, which mainly included samples from Cape Cod. At the regional scale, the eastern cottontail’s first MEMGENE axis showed a split in the north/south cardinal direction that separated the samples into two approximately equal groups. The divide occurred at the western border of Rhode Island north through Worcester, Massachusetts. At the Cape Cod scale, both species showed an east/west divide. The eastern cottontail’s first MEMGENE axis showed three groups with the major split (black and white triangles) occurring near the town of Centerville. The size of the white triangles separated into two groups with a divide near the town of South Dennis. New England cottontail did not have any MEMGENE axes for the West region. Eastern cottontail’s first MEMGENE axis in the West region showed a split in the north/south cardinal direction. Both the New England cottontail and the eastern cottontail’s first MEMGENE axis in the East subregion showed a split into two groups that were divided by the Thames River in Connecticut.

### Environmental Variation Analyses

A map of each environmental variable is shown in Supplementary Appendix S3. The optimized number of trees (ntree) for every analysis was 500. The percent of variation explained by the New England cottontail *randomForest* analyses were 25, 19, and 65% for the Cape Cod, West, and East subregions, respectively. The percent of variation explained by the eastern cottontail *randomForest* analyses were 38, 62, and 41% for the Cape Cod, West, and East subregions, respectively. The two measures for the most important variables in the *randomForest* analyses agreed in eight out of 12 instances (Table 3). The most important environmental variables varied by subregion with some similarities among subregions and species. The variable *distance to shrub cover* was identified as a top two variable the most times out of all variables (4 out of 6). Elevation was the only other variable that placed in the top two more than one time (2 out of 6).

**TABLE 3:** Random Forest average percent increase in mean squared error (IncMSE) and node purity (IncNP) with standard deviation (SD) results for A) New England cottontail (NEC) and B) eastern cottontail (EAC) at the subregional levels, Cape Cod (CC), West, and East. The environmental variables included aspect, elevation based on a digital elevation model, distance to medium and high development (dist_dev), distance to roads (dist_road), distance to shrub cover (dist_shrub), distance to forest and herbaceous wetland (dist_wetlands), and forest height (for_height).

A						
Variable	CC IncMSE (SD)	IncNP (SD)	West IncMSE (SD)	IncNP (SD)	East IncMSE (SD)	IncNP (SD)
Aspect	30.676 (3.135)	0.173 (0.000)	8.029 (0.501)	0.031 (0.002)	21.820 (0.948)	0.076 (0.003)
Elevation	32.608 (1.580)	0.182 (0.002)	12.323 (0.680)	0.019 (0.001)	14.277 (0.664)	0.068 (0.000)
dist_dev	51.149 (9.147)	0.165 (0.001)	10.589 (0.505)	0.030 (0.000)	12.693 (0.334)	0.081 (0.005)
dist_road	52.925 (4.488)	0.158 (0.001)	6.569 (0.545)	0.019 (0.001)	20.116 (1.001)	0.109 (0.005)
dist_shrub	59.475 (2.221)	0.157 (0.002)	11.960 (0.329)	0.042 (0.002)	14.294 (0.429)	0.057 (0.002)
dist_wetland	58.649 (4.865)	0.160 (0.002)	10.321 (0.182)	0.018 (0.001)	12.083 (0.754)	0.035 (0.001)
forest_height	42.367 (6.605)	0.168 (0.003)	9.422 (0.311)	0.027 (0.001)	14.794 (0.588)	0.040 (0.001)

B						
Variable	CC IncMSE (SD)	IncNP (SD)	West IncMSE (SD)	IncNP (SD)	East IncMSE (SD)	IncNP (SD)
Aspect	43.261 (0.260)	0.095 (0.001)	17.002 (1.020)	0.014 (0.000)	30.838 (1.293)	0.046 (0.000)
Elevation	56.420 (2.355)	0.117 (0.002)	35.460 (0.271)	0.028 (0.001)	65.211 (2.329)	0.090 (0.001)
dist_dev	43.599 (1.123)	0.095 (0.001)	27.711 (1.704)	0.021 (0.000)	57.276 (3.806)	0.092 (0.001)
dist_road	43.500 (4.146)	0.097 (0.001)	25.692 (1.744)	0.023 (0.001)	51.898 (2.210)	0.066 (0.000)
dist_shrub	45.923 (1.961)	0.101 (0.001)	41.834 (0.984)	0.058 (0.002)	46.910 (2.069)	0.066 (0.001)
dist_wetland	41.278 (1.490)	0.090 (0.002)	28.709 (1.201)	0.029 (0.001)	46.414 (1.592)	0.061 (0.001)
forest_height	37.797 (1.370)	0.085 (0.001)	36.629 (2.278)	0.051 (0.001)	43.665 (1.578)	0.063 (0.001)

**FIGURE 5:**
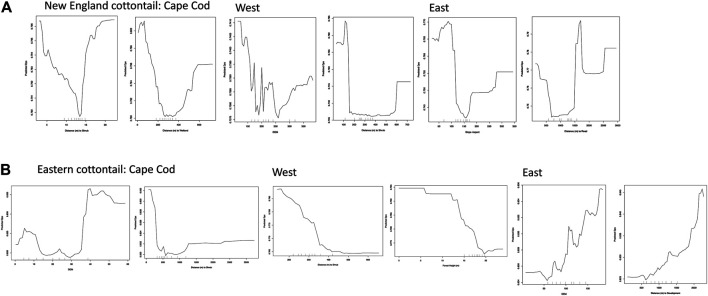
Random Forest partial dependence plots created using the *partialDependence* function in R version 3.6.2 (R Core Team 2019) for **(A)** New England cottontail (NEC) and **(B)** eastern cottontail (EAC) at the subregional levels, Cape Cod, West, and East. The predicted proportion of shared alleles (Dps) are relative to an increase in a given environmental variable. The environmental variables included aspect, digital elevation model (DEM), distance to medium and high development, distance to roads, distance to shrub cover, distance to forest and herbaceous wetland, and forest height.

The partial dependence plots visually show the relationship between increasing values of a given environmental variable on the x-axis and an increasing measure of gene flow (Dps) on the y-axis. The slope of the partial dependence plots for the top two environmental variables for each region was as predicted for the most part (Figure 5). The predicted Dps values always decreased as the distance to shrub variable increased for both species in the Cape Cod and West subregions. However, the relationship was non-linear for three of the four plots. We did not have an *a priori* expectation for an impact of elevation or slope aspect on Dps, but in both cases the influence pattern was non-linear for New England cottontail. However, for eastern cottontail the relationship between elevation and Dps was linear and the proportion of shared alleles increased as elevation increased. We expected distance to road and development to have a negative influence on gene flow with lower distance values correlating to lower Dps values. The relationship was non-linear for distance to road for New England cottontail, but linear in the expected direction for distance to development for eastern cottontail. The distance to wetlands variable was unexpectedly non-linear, but this variable was not identified as a top variable by both measures for New England cottontail on Cape Cod (Table 3). The distance to forest was as expected for eastern cottontail with higher forest heights correlating with lower Dps values (Figure 5).

## Discussion

### Specialist-Generalist Variation Hypothesis

In this study, we used spatially explicit, individual-based comparative approaches to test various predictions of the SGVH in two sympatric cottontail species and identify environmental variables that influence gene flow in each species. Our first hypothesis predicted that New England cottontail would have lower genetic diversity and Ne values than eastern cottontail. Numerous previous comparisons of related taxa have shown that threatened species have lower genetic diversity than conspecifics that are non-threatened (Frankham et al., 2017). Surprisingly, average genetic diversity was the same or higher for New England cottontail compared to eastern cottontail. However, we did identify more than four times the number of SNPs for eastern cottontail than New England cottontail. The lower than expected genetic diversity values for eastern cottontail could be explained if the species endured bottlenecks and/or founder effects due to their introduction to the Northeastern US. We don’t expect this to be the case on mainland areas because eastern cottontail have an extraordinarily high number of mitochondrial DNA haplotypes (Sullivan et al., 2019) and an incredibly high level of immunogenetic diversity (Neil, 2020). Interestingly, eastern cottontail from Cape Cod had the highest inbreeding value, which could indicate animals from that region have suffered the negative impacts of a bottleneck and/or founder effects. A notable potential barrier to gene flow is the Cape Cod canal, which was built in the early 1900s (https://www.nae.usace.army.mil/Portals/74/docs/Recreation/CCC/Brochures/Canal_Overview_Trifold.pdf) before the documentation of eastern cottontail on Cape Cod (Litvaitis et al., 1997). In all likelihood, eastern cottontail were transported to Cape Cod in potentially low numbers. Another island south of Cape Cod where both cottontail species could have been introduced is Nantucket Island. Interestingly, samples from both species from Nantucket Island clustered with samples from the East subregion, which could indicate the animals originated from that area. However, the spatial MEMEGENE analysis grouped the New England cottontail samples from Nantucket Island with samples from the Cape Cod subregion, but the eastern cottontail samples from Nantucket Island mainly grouped with samples from southern Rhode Island. In general, the inbreeding values for both species were comparable with overlapping standard error ranges for all comparisons. We had expected New England cottontail to have higher inbreeding values due to their decline in distribution. The similar inbreeding values between the two species is an encouraging result for New England cottontail and the lower values could be maintained due to juvenile male dispersal. Two major genetic concerns in small populations are loss of genetic diversity and increased inbreeding levels (Frankham et al., 2017) and New England cottontail have maintained relatively good values for both measures.

Our genetic diversity results are similar to those of Fenderson et al. (2011), who found higher levels of Ho and unbiased He in the western region of New England cottontail’s range based on analyses with microsatellite markers. Fenderson et al. (2011) found drift had impacted New England cottontail populations. When Ho is less than He, this can be due to drift fixing alleles in a small population (Frankham et al., 2017), which is called the Wahlund effect (Wahlund, 1928). We found evidence for drift impacting nearly all subregions for New England cottontail, except for Cape Cod. Fenderson et al. (2011) also found New England cottontail from Cape Cod have gone through a genetic bottleneck, but our results don’t support this finding. Unexpectedly, all the eastern cottontail subregions also showed evidence of the Wahlund effect. Eastern cottontail is nearly an ubiquitous invasive species on the landscape and we did not expect drift to be impacting their populations.

The Ne estimates for eastern cottontail were generally higher than New England cottontail Ne values, but only one region showed a significant difference (i.e., non-overlapping standard error estimates). In general, these results support the second part of our first hypothesis. However, we expected a more drastic difference between the two species, given the known decline of New England cottontail and the geographic restriction of the species (Litvaitis et al., 2006; Rittenhouse and Kovach, 2021). Our Ne estimates for New England cottontail also are different from Fenderson et al. (2011). We found that New England cottontail from the Cape Cod subregion had the highest Ne and New England cottontail from the East subregion had the lowest Ne. Overall, the Ne estimates for the native species in these subregions are lower than the minimum Ne of 300 recommended for lagomorphs (Newmark, 1995; Cheeseman et al., 2019). For the invasive eastern cottontail, the upper bound confidence value for Ne was above the 300 threshold in two out of the three subregions, suggesting that this species is more likely to persist into the near future. However, even though our estimates were based on a higher number of markers than previous studies, these reflect neutral levels of genetic diversity and we have not estimated adaptive variation. The higher than expected genetic diversity values for New England cottontails is encouraging for the long term genetic health of the species and based on the Ne estimates, the Cape Cod population will have the slowest decline in neutral genetic diversity.

Genetic diversity and population structure of species inhabiting the same range depend on fine-scale responses to environmental factors (Berkman et al., 2015 and references therein) and demographic effects (Frankham et al., 2017). Our second hypothesis predicted that New England cottontail gene flow will be more restricted than eastern cottontail gene flow and environmental variables will explain less of the genetic differentiation among eastern cottontail. At a regional level, our hypothesis was supported with the population clusters of New England cottontail more distinct than eastern cottontail. This result is not surprising given the disjunct distribution of New England cottontail and supports previous research (Fenderson et al., 2011; Neil 2020). However, our spatially explicit analyses at the subregional levels showed more geographic areas of restricted gene flow for eastern cottontail than New England cottontail. Only the EEMs analysis for New England cottontail on Cape Cod showed a stronger reduction in gene flow compared to eastern cottontail gene flow. The most complex subregion pattern for eastern cottontail was the East subregion that displayed multiple areas of restricted gene flow. Interestingly, the regional EEMS analysis pattern for both species showed the same general pattern. This could indicate similar major barriers to cottontail gene flow in those areas. However, eastern cottontail showed more areas of stronger restricted gene flow compared to New England cottontail. The sampling distribution for New England cottontail was more patchy compared to eastern cottontail due to their disjunct distribution, which could explain the difference in pattern between the species.

We found a significant isolation by distance pattern for eastern cottontail at the regional level. Neil (2020) did not find a significant pattern at the regional level for eastern cottontail, but the analyses were at the population level. The analysis of isolation by distance at the individual level can be more sensitive to detection than analyses at the population level (Prunier et al., 2013). At an individual level, Prunier et al. (2013) recommends to use partial Mantel tests to remove comparisons of individuals within a population. We selected our subregions to each include samples from areas pre-identified as populations for both species, so comparisons were all within a population. This was done in an attempt to remove the influence of population structure and isolation by distance on our analyses identifying environmental variables influencing each species’ gene flow. The only subregion that we did not detect a pattern of isolation by distance was Cape Cod for New England cottontail. New England cottontail in this subregion had the highest Ne for the species and may represent a more continuous population, which could explain why we did not detect isolation by distance.

We predicted the MEMGENE adjusted R-squared values would be higher for New England cottontail compared to eastern cottontail because we expected the environment to have a stronger impact in limiting New England cottontail gene flow. Eastern cottontail are thought to be better dispersers than New England cottontail and their movements less restricted by the environment (Litvaitis et al., 2008). The adjusted R-squared values for New England cottontail were higher for two of four comparisons (regional and East), but comparisons can be influenced by uneven sample size and the demographic histories of the compared species (Hein et al., 2021). Two areas showed a similar MEMGENE eigenvector map pattern for both species at the Cape Cod and East subregions. The similar east/west divide on the Cape Cod subregion for both species is somewhat puzzling because there are no apparent environmental barriers. The subdivision of eastern cottontail on Cape Cod also was detected by Neil (2020) at the regional scale and our non-spatial DAPC analysis. The elevation on Cape Cod increases as you move northwest and elevation was identified as an important environmental variable for eastern cottontail in that subregion. Elevation can influence the type of vegetation that grows in an area, but the range of elevation values on Cape Cod is only from sea level to about 93 m above sea level. However, the upper Cape Cod region in the Northwest has vegetation that is more similar to mainland Massachusetts and has deciduous forests (Harper, 1921). The environmental barrier could be the vegetation transition in that area on Cape Cod. The MEMGENE analysis for both species in the East subregion showed a separation of the samples in the southeast corner, which are separated by the Thames River that likely serves as a major barrier to gene flow in both species. The DAPC assignment for both cottontail species in the East subregion also showed this same split with samples from the southeast corner separated from the rest of the samples. The percent of variation explained by the Random Forest analyses also was not consistently higher for New England cottontail compared to eastern cottontail, which was counter to our *a priori* expectation.

Our two hypotheses were not fully supported by our results. Thus, our results only provide partial support for the SGVH because our expected outcomes were inconsistent. Prior to our study, the SGVH had yet to be explicitly applied to vertebrates. However, there are examples of vertebrates where the SGVH is supported, which include cottontails (Berkman et al., 2015) and felids (Janecka et al., 2016). Expected effects on genetic variability and population structure were observed in ocelot and bobcat that are a specialist-generalist species pair sharing the same range, where the dichotomy between habitat specialists and habitat generalists was observed at genetic variability and population structuring levels (Janecka et al., 2016). Support for the SGVH also has been found for numerous flying invertebrates. Zayed et al. (2005) found the expected pattern of increased genetic differentiation in a specialist bee compared to a generalist bee. Taylor et al. (2020) compared the mitochondrial DNA genetic structure of a widespread mayfly to two endemic mayfly species and found a lack of population structure in the habitat generalist and the presence of population structure for the two habitat specialist species. Maresova et al. (2019) found the expected outcome predicted by the SGVH with higher genetic diversity and a lack of structure for the generalist butterfly, while the specialist butterfly showed a stronger pattern of geographic structure.

### Influence of the Environment on Gene Flow

In general, our predicted influence of a given environmental variable on genetic differentiation matched our expectations. Natural environmental variables that were important factors influencing cottontail gene flow included shrub, wetlands, and forest height. Distance to shrub was identified as a top important environmental variable the highest number of times, two times for each species in a subregion. The influence of distance to shrub was mostly as expected with a decreasing proportion of shared allele values as the distance to shrub habitat increased. The relationship was non-linear for New England cottontail on Cape Cod, but the two importance measures were inconsistent for that environmental variable. Distance to shrub habitat was expected to have a stronger influence on New England cottontail than eastern cottontail because New England cottontail are early successional habitat specialists. One of the main habitat characteristics of early successional habitat is dense shrub vegetation. Our results support the importance of shrub habitat in facilitating cottontail gene flow and support previous research that has found both species have high detection rates in this type of habitat (Mayer et al., 2018; Shea et al., 2019). Distance to wetlands was only identified as an important variable for New England cottontail, but this variable was only identified as important by one of the two importance measures. Both cottontail species do not avoid wetlands, at least during the winter (Buffum et al., 2015), and this type of habitat overlaps with shrub. Forest height was predicted to have a stronger influence on eastern cottontail than New England cottontail and this environmental variable was only identified as an important variable for eastern cottontail. New England cottontails have been found in higher canopy cover and closure than eastern cottontail (Buffum et al., 2015; Mayer et al., 2018). Closed canopies could serve as a barrier to eastern cottontail gene flow.

Anthropogenic factors that influenced cottontail gene flow included development and roads. We expected distance to development to have a stronger impact on New England cottontail gene flow because eastern cottontail can inhabit urban areas. However, the variable was only identified as important for eastern cottontail in one subregion and the influence of the variable was as expected with a higher proportion of shared allele values as the distance to development increased. Roads have been identified as both facilitators and inhibitors of New England cottontail gene flow (Fenderson et al., 2014; Amaral et al., 2016). The influence of distance to roads for New England cottontail was non-linear, which may reflect the dual nature of roads both facilitating and inhibiting gene flow. We didn’t expect elevation to influence the gene flow of either species, but it was identified as an important environmental variable for eastern cottontail. The influence of elevation was both non-linear and linear, which would make it difficult to base management decisions on this environmental variable.

Extensive efforts to create New England cottontail habitat have been conducted by state and federal biologists (Fuller and Tur, 2012). The creation of early successional habitat is a major focus, which unfortunately will benefit the gene flow for both species. The main environmental variable we identified that could limit eastern cottontail gene flow is forest height. Cheeseman et al. (2018) also found that eastern cottontail did not occupy areas with high canopy closure, but New England cottontail would occupy those areas when there was a high prevalence of eastern cottontail. The selection of sites to manage the habitat for New England cottontail could include areas that have surrounding mature forest. The mature forest could act as a barrier to eastern cottontail infiltration if New England cottontail are released in the managed area. However, if the New England cottontail disperse from the managed habitat to the areas with more closed canopy forests, they could be creating an ecological trap if their survival is lower in that type of habitat (Cheeseman et al., 2021).

The variable results of which environmental variable was the most influential in affecting a given species’ gene flow demonstrates that location matters and makes it difficult to generalize results from one area of a species’ range. Fenderson et al. (2014) also found a differing influence of environmental variables on New England cottontail gene flow in the upper Northeastern part of their range that differed based on landscape configuration. Thus, this pattern holds range wide for New England cottontail and the three subregions we analyzed for eastern cottontail.

### Future Directions

The evolutionary potential is not always different between specialists and generalists. Martinossi-Allibert et al. (2017) showed in wild birds no significant effect of habitat specialization on any of the quantitative genetic estimators, except that generalists have higher heterozygosity, mainly due to their larger population size. Our analyses were based on neutral genetic markers and to assess evolutionary potential, adaptive genetic variation needs to be measured for each species. Interestingly, we identified more potentially adaptive SNPs for New England cottontail (*n* = 57) compared to eastern cottontail (*n* = 41), but additional analyses would be needed to confirm that these SNPs are adaptive. Adaptive SNPs also are meaningless unless they can be associated with a particular environment, geographic location, or phenotype.

The analysis of adaptive markers could help identify evolutionary significant units for New England cottontail, as well as, the best source populations for reintroductions that are not too divergent from the target recipient population to avoid outbreeding depression (Funk et al., 2012). Conversely, the interbreeding of animals from different populations can increase their genetic diversity (i.e., heterosis) and the risk of the animals suffering from outbreeding depression is typically low (Frankham et al., 2011). Currently, the New England cottontail conservation program has been interbreeding animals from throughout their range in a captive setting to maximize their genetic diversity because the risk of outbreeding depression is thought to be low due to a lack of risk factors identified by Frankham et al. (2017). However, a direct analysis of New England cottontail adaptive genetic variation would be needed to determine if New England cottontail from different geographic areas retain unique adaptive differences. Maximizing New England cottontail adaptive genetic variation also is important for their resistance to diseases. Recently, concerns about the spread of rabbit hemorrhagic disease virus, type 2 have increased as it has spread from the west coast of the US to the east as far as New York City (https://www.aphis.usda.gov/animal_health/downloads/rhdv2.pdf). Tularemia, a bacterial disease, also has recently been detected in an island population of New England cottontail. Retaining New England cottontail adaptive potential will be critical to allow them the best chance possible to survive these disease challenges and other threats, such as climate change. The identification of environmental variables that facilitate and inhibit gene flow also will help predict how diseases will be able to spread among and within both species. Analyzing populations throughout the species’ range will be critical to ensure informed management decisions.

## Data Availability

The cleaned fastq files are deposited in NCBI’s SRA database under BioProject accession number PRJNA736852.
