# Supplementary material for: Location and Species Matters: Variable Influence of the Environment on the Gene Flow of Imperiled, Native and Invasive Cottontails
Source: Front Genet. 2021 Sep 29;12:708871. doi: 10.3389/fgene.2021.708871 (PMC8511500; doi:10.3389/fgene.2021.708871)

# Supplementary Material

Appendix I. Tests for isolation by distance at an individual level by comparing the genetic difference matrices estimated using the function *bed2diffs_v2* (Petkova et al. 2016) to geographic distance using GenAlEx 6.502 (Peakall and Smouse 2006; Peakall and Smouse 2012) with 9,999 permutations and an alpha value of 0.05. Tests were conducted at regional and subregion scales for New England cottontail (NEC; *Sylvilagus transitionalis*) and eastern cottontail (EAC; *S. floridanus*).

A) NEC (regional; n=191)

B) NEC (Cape Cod; n=114)

C) NEC (West; n=31)

D) NEC (East; n=31)

E) EAC (regional; n=274)

F) EAC (Cape Cod; n=70)

G) EAC (West; n=29)

H) EAC (East; n=29)

Appendix II. Discriminate Analysis of Principal Components (DAPC) (Jombart et al. 2010) plots of the first two axes created using the R package *adegenet* (Jombart, 2008) for each subregion. Also shown are the discriminant function plots that show the meaningful number of clusters for a given subregion and spatial distribution of two clusters for eastern cottontail in the West and East subregions.

1. NEC (Regional)

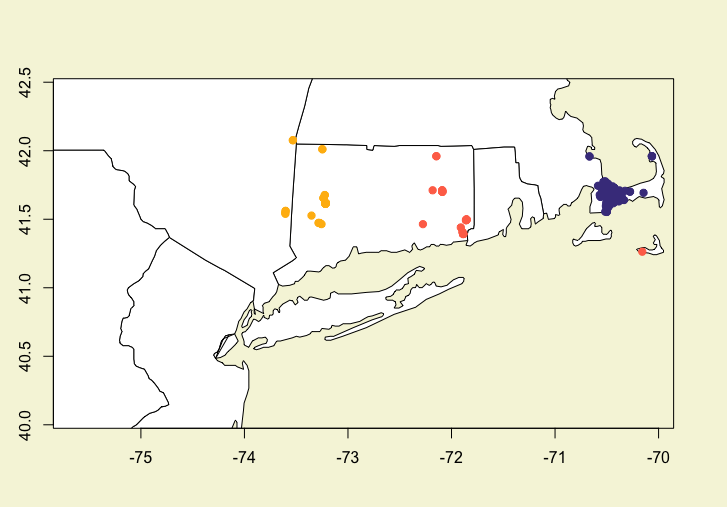


1. EAC (Regional)

1. NEC (Cape Cod)

1. EAC (Cape Cod)

1. NEC (West)

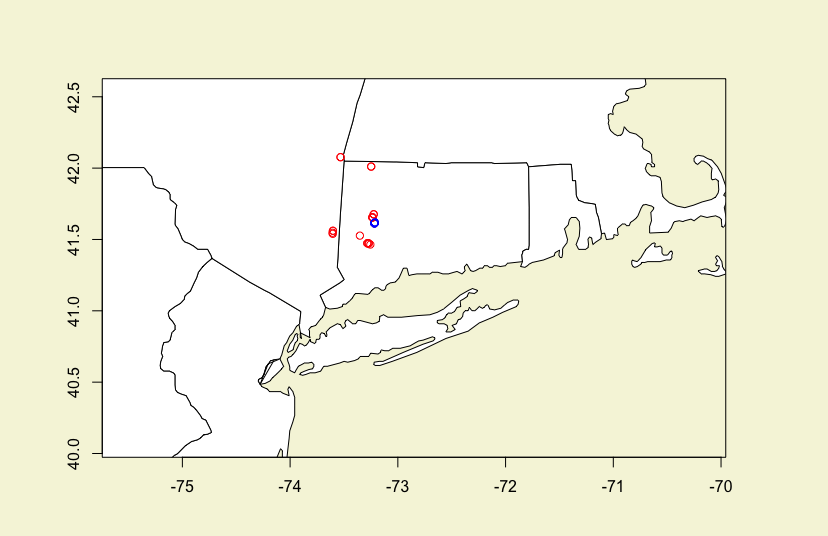


1. EAC (West)

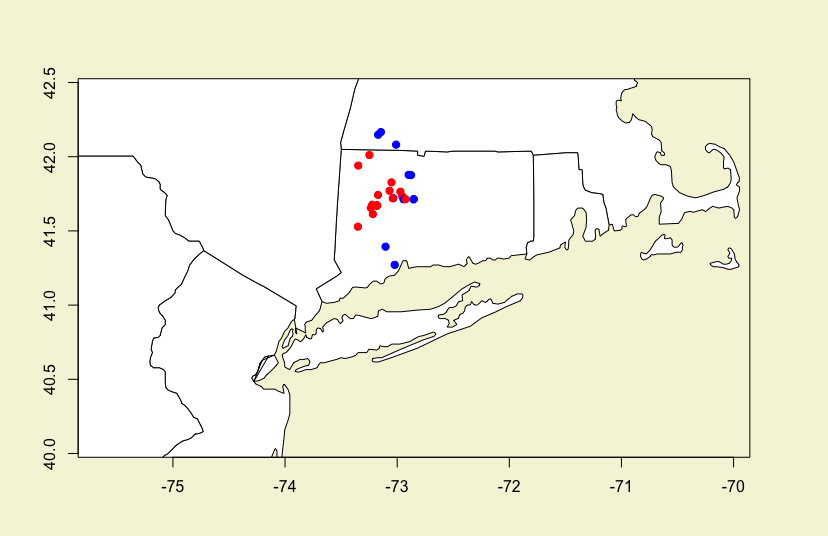


1. NEC (East)

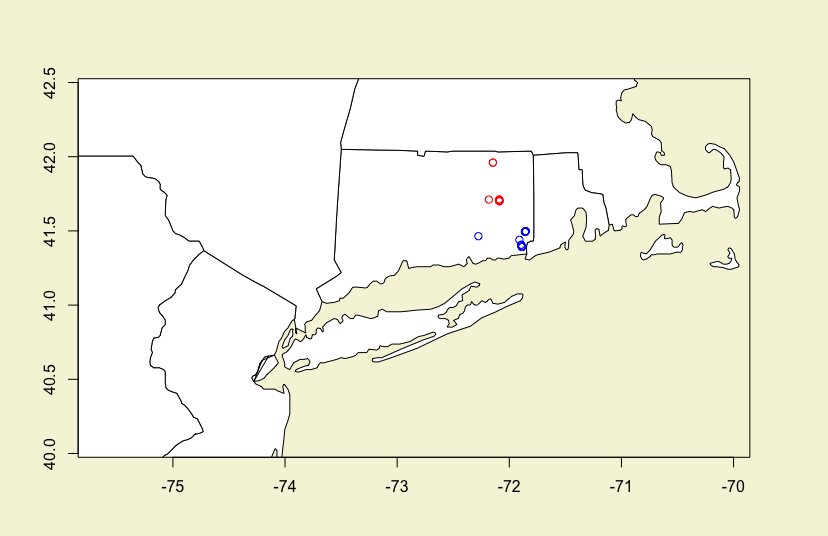


1. EAC (East)

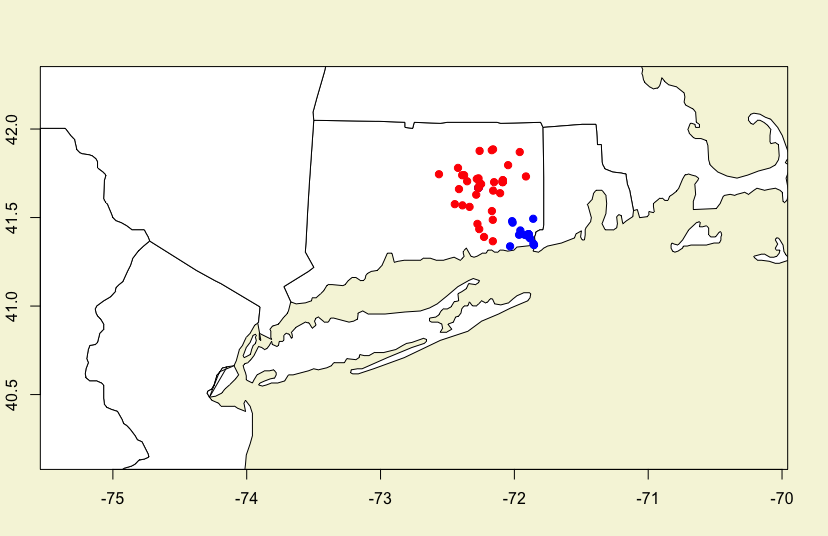

Supplement: Supplementary file 1 [file Table1.docx]
