# Supplementary material for: Location and Species Matters: Variable Influence of the Environment on the Gene Flow of Imperiled, Native and Invasive Cottontails
Source: Front Genet. 2021 Sep 29;12:708871. doi: 10.3389/fgene.2021.708871 (PMC8511500; doi:10.3389/fgene.2021.708871)
Supplement: Supplementary file 2 [file Table2.docx]

Appendix III. Random Forest geographic information system environmental variables. Distances are in meters.

1. Cape Cod

Aspect Digital Elevation Model (DEM)


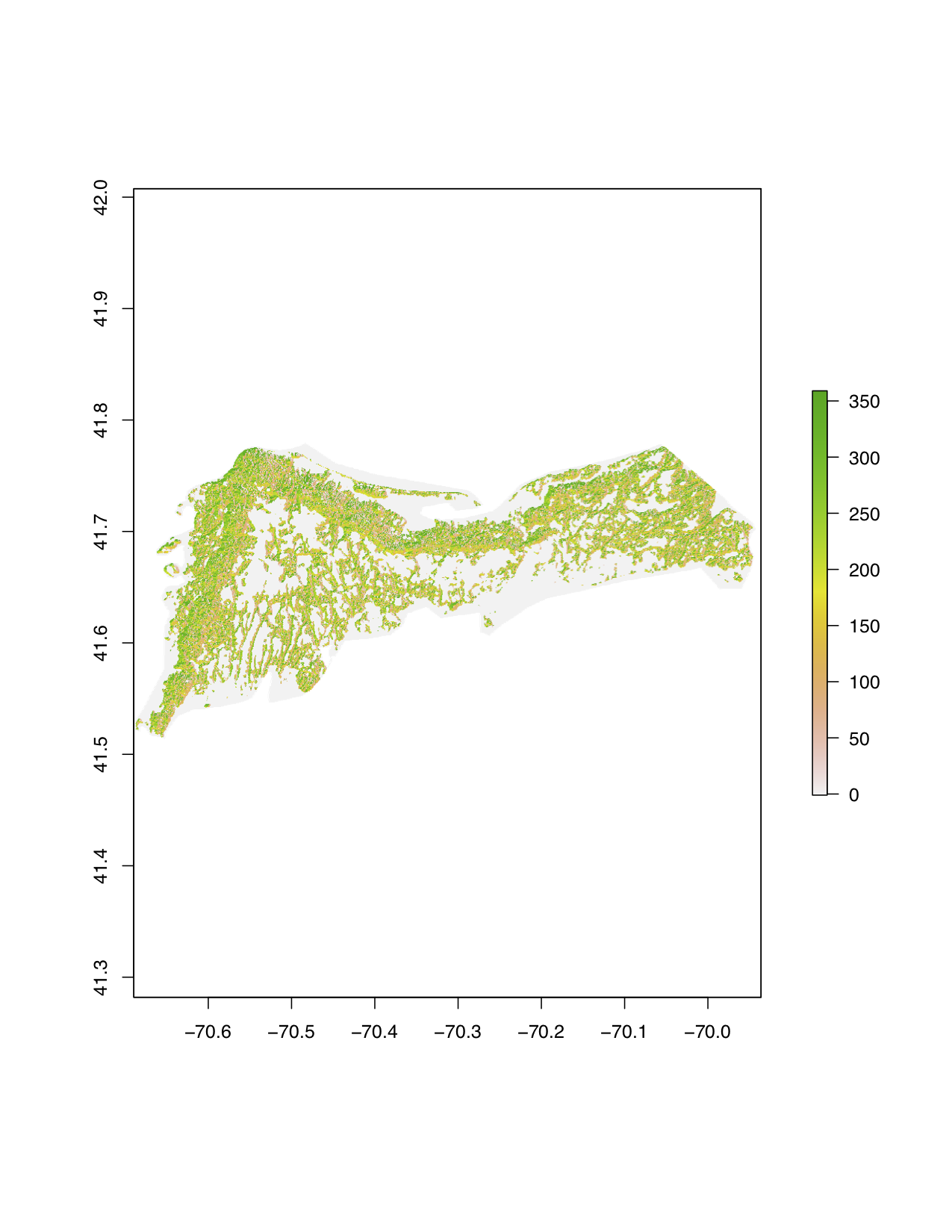

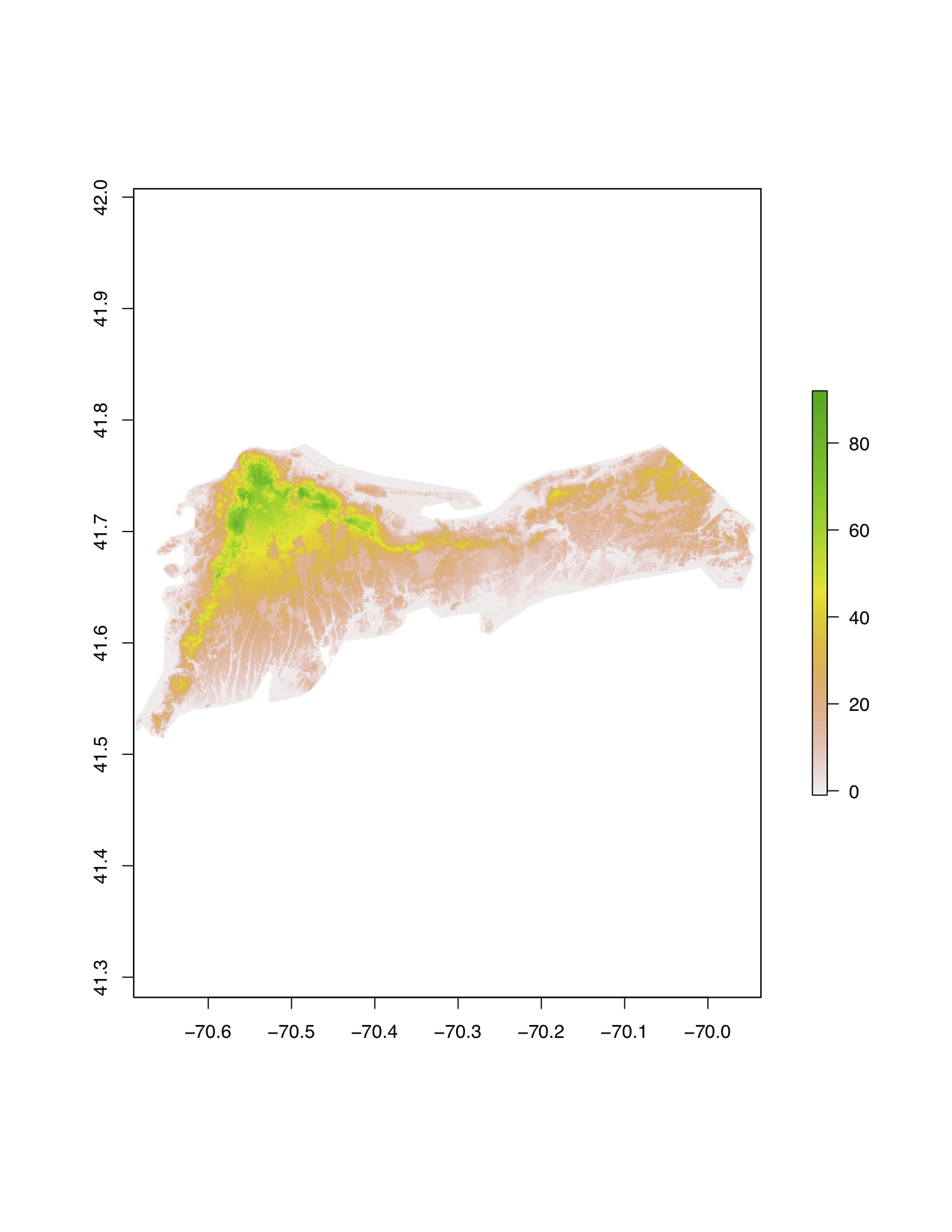


Distance to Development Distance to Road


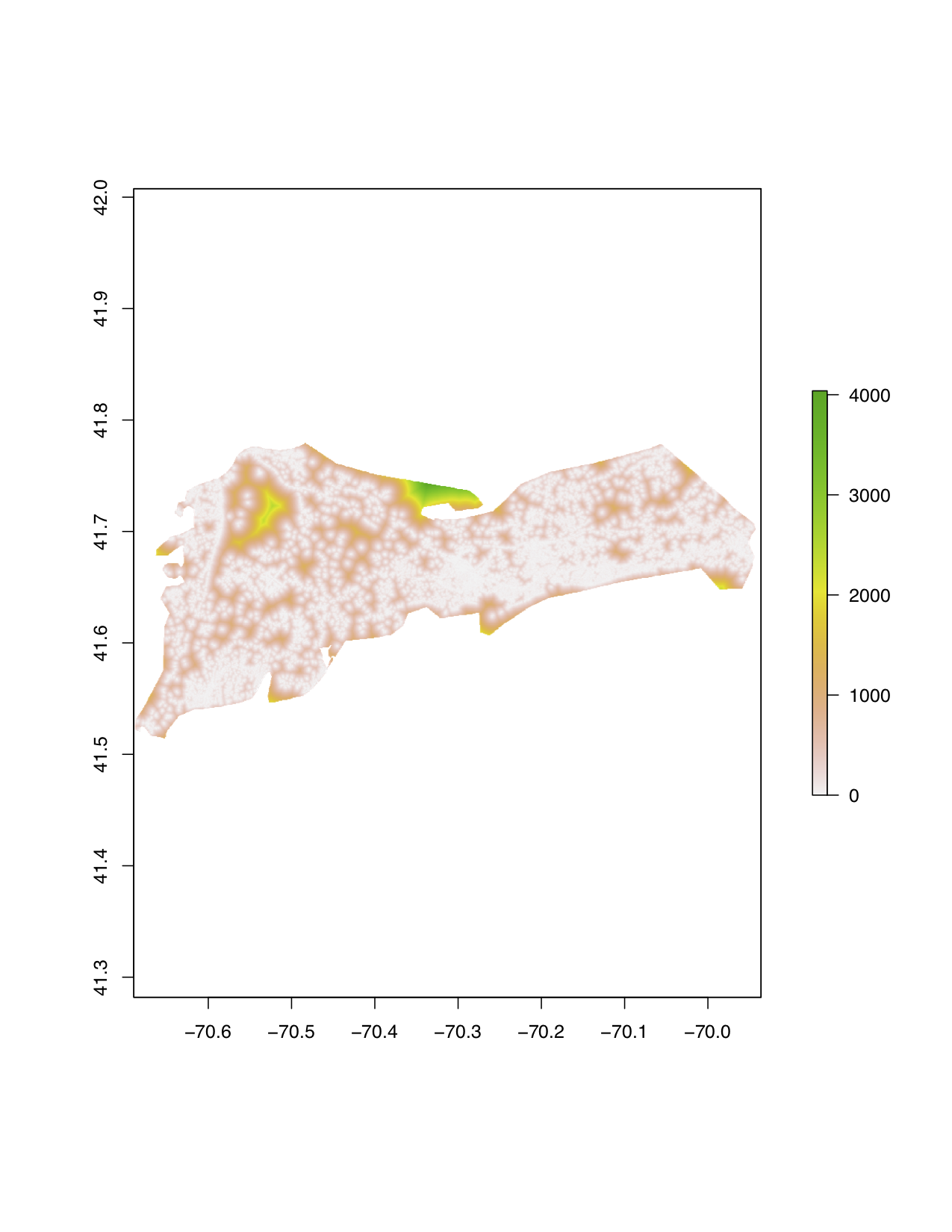

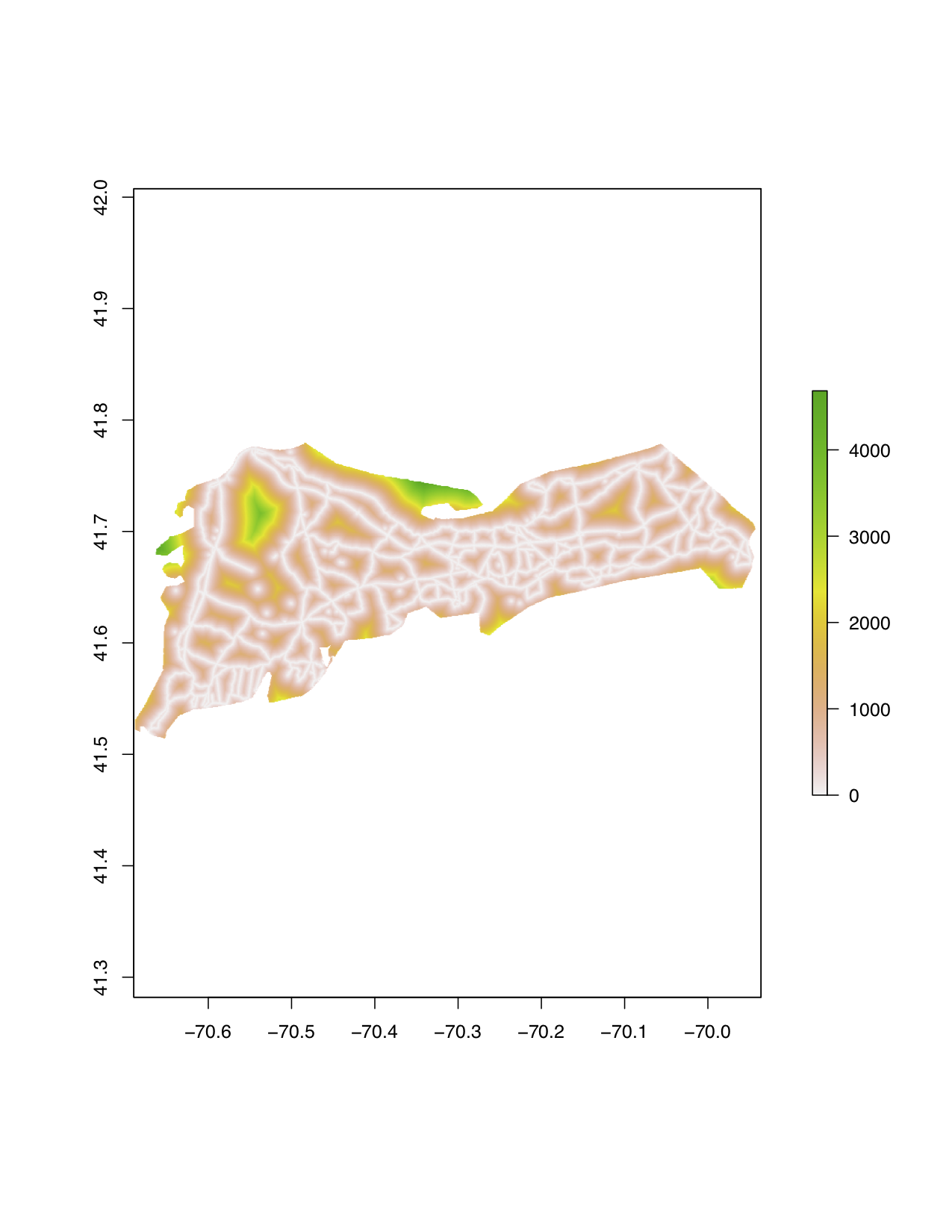


Distance to Shrub Cover Distance to Wetlands


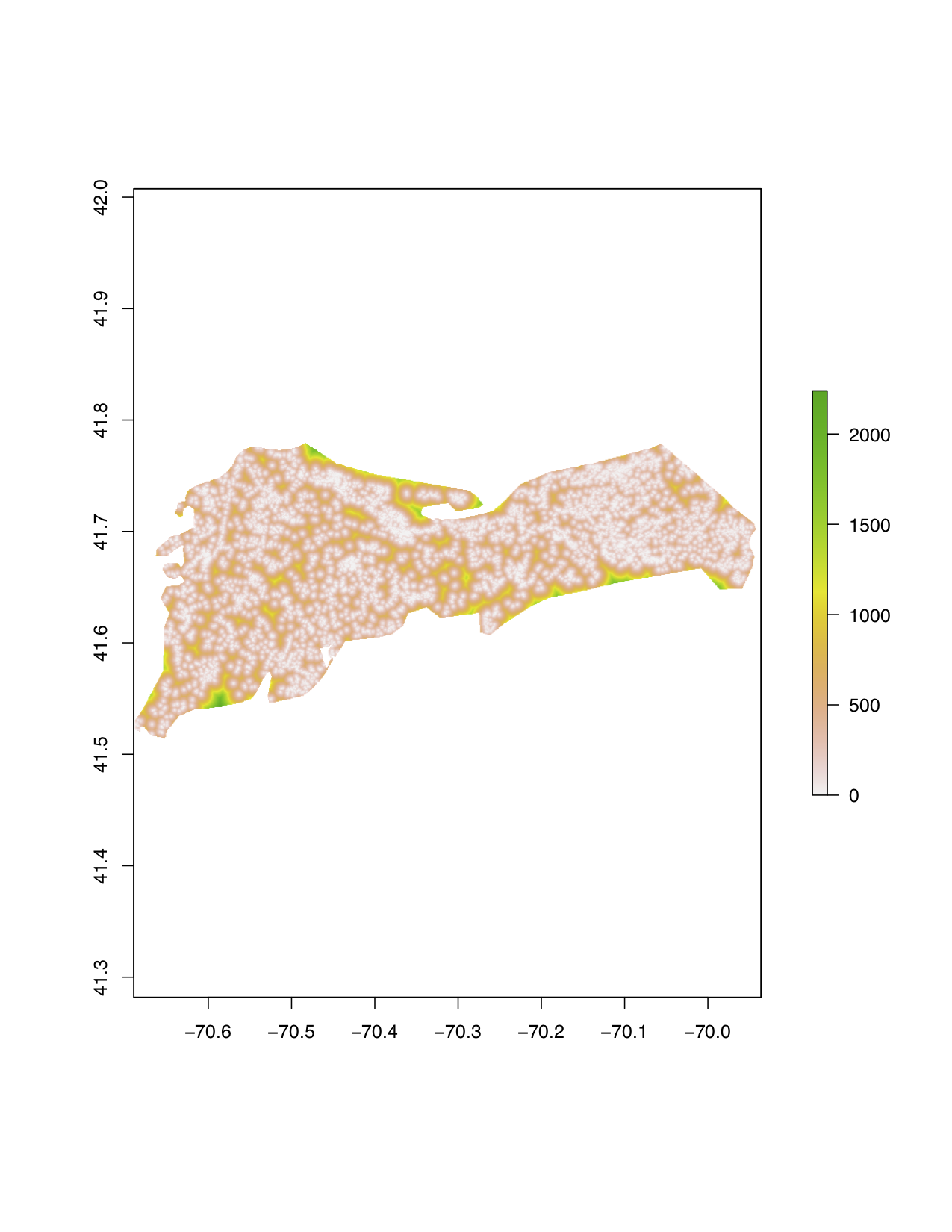

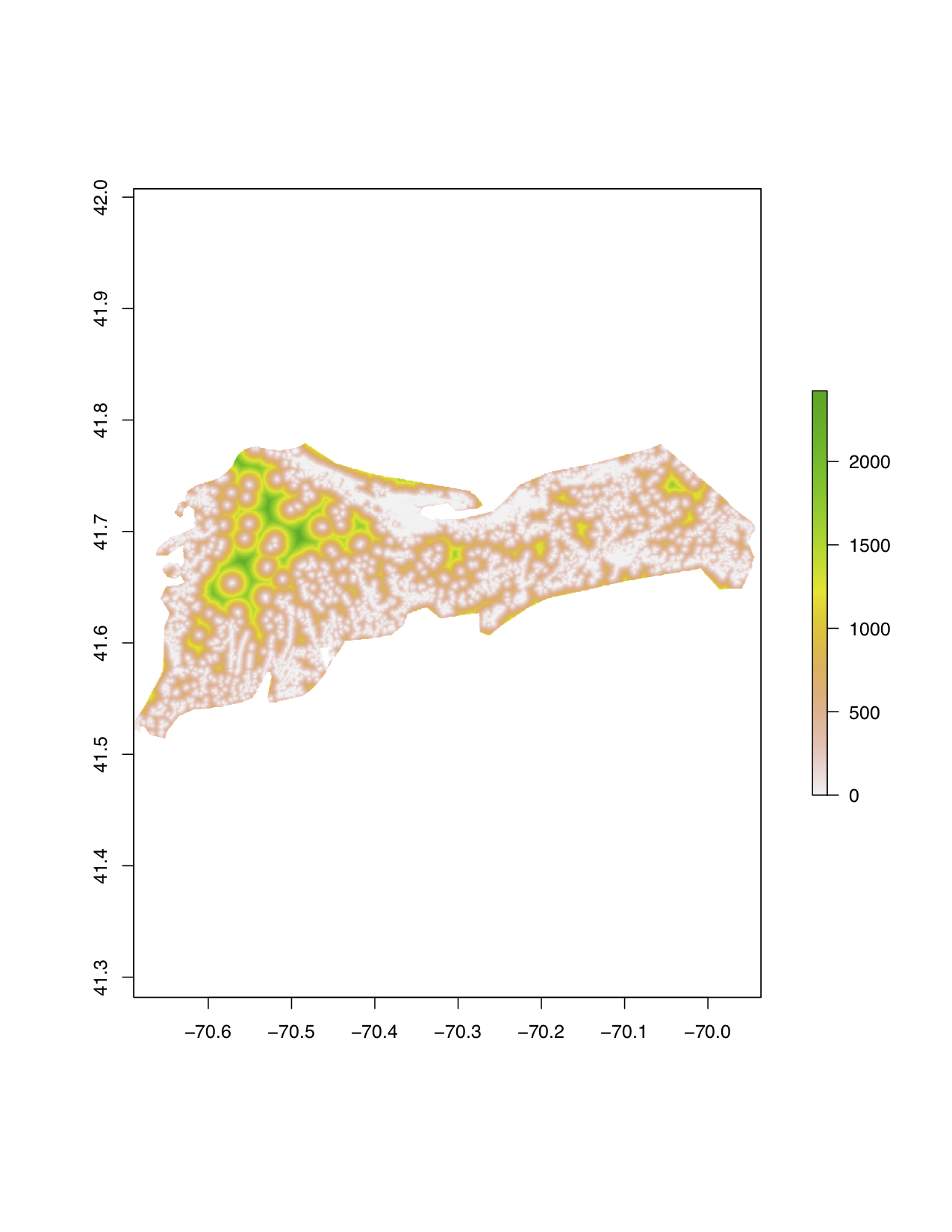


Forest Height


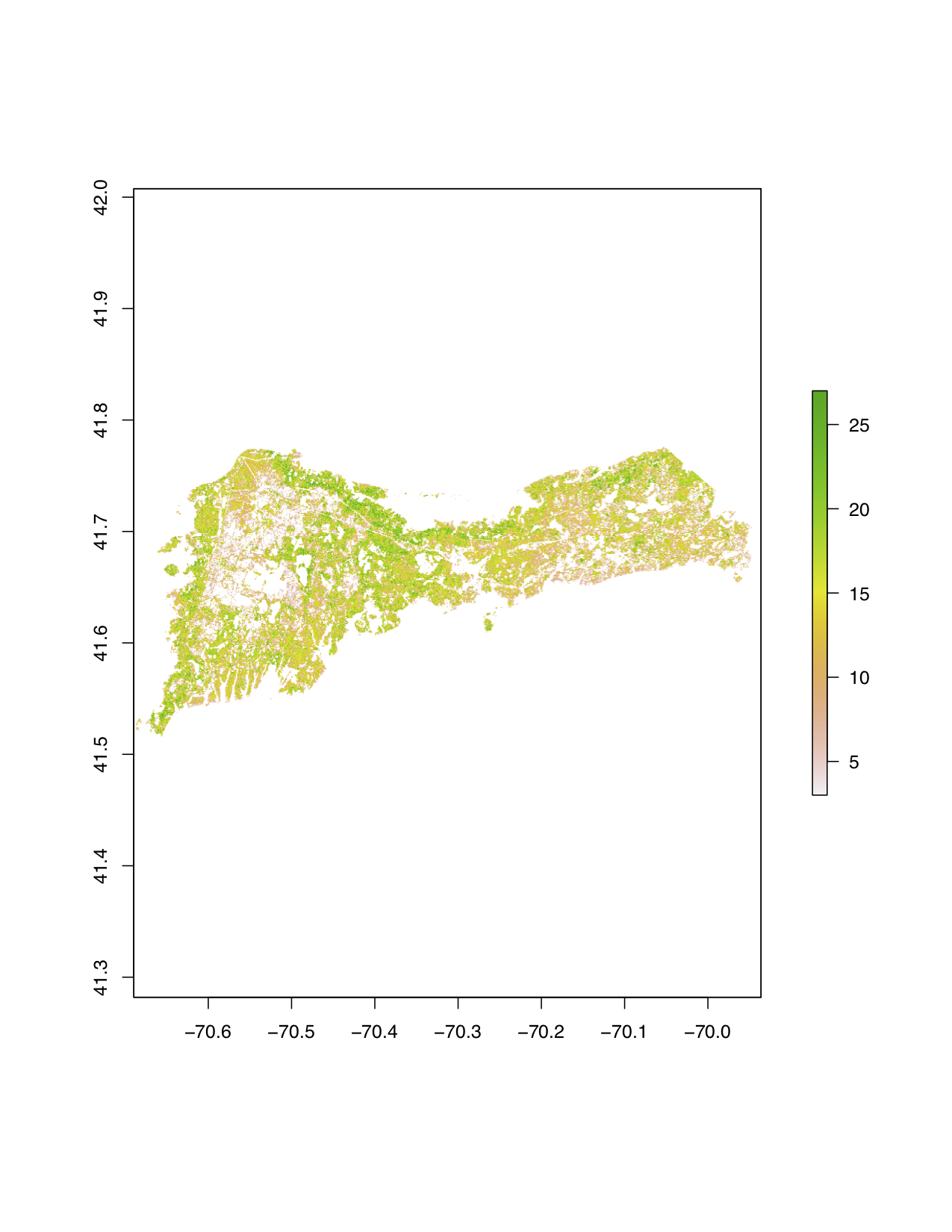


1. West

Aspect DEM


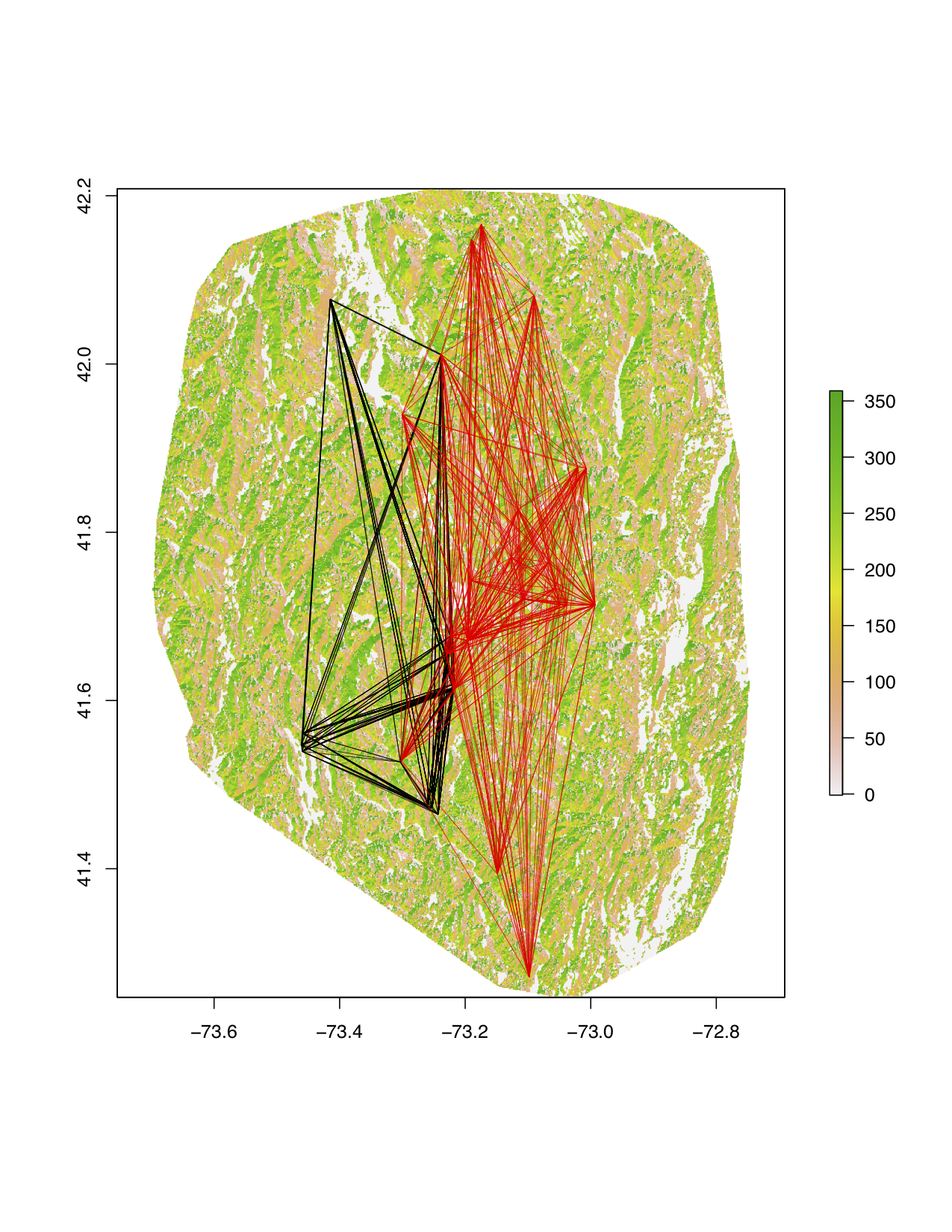

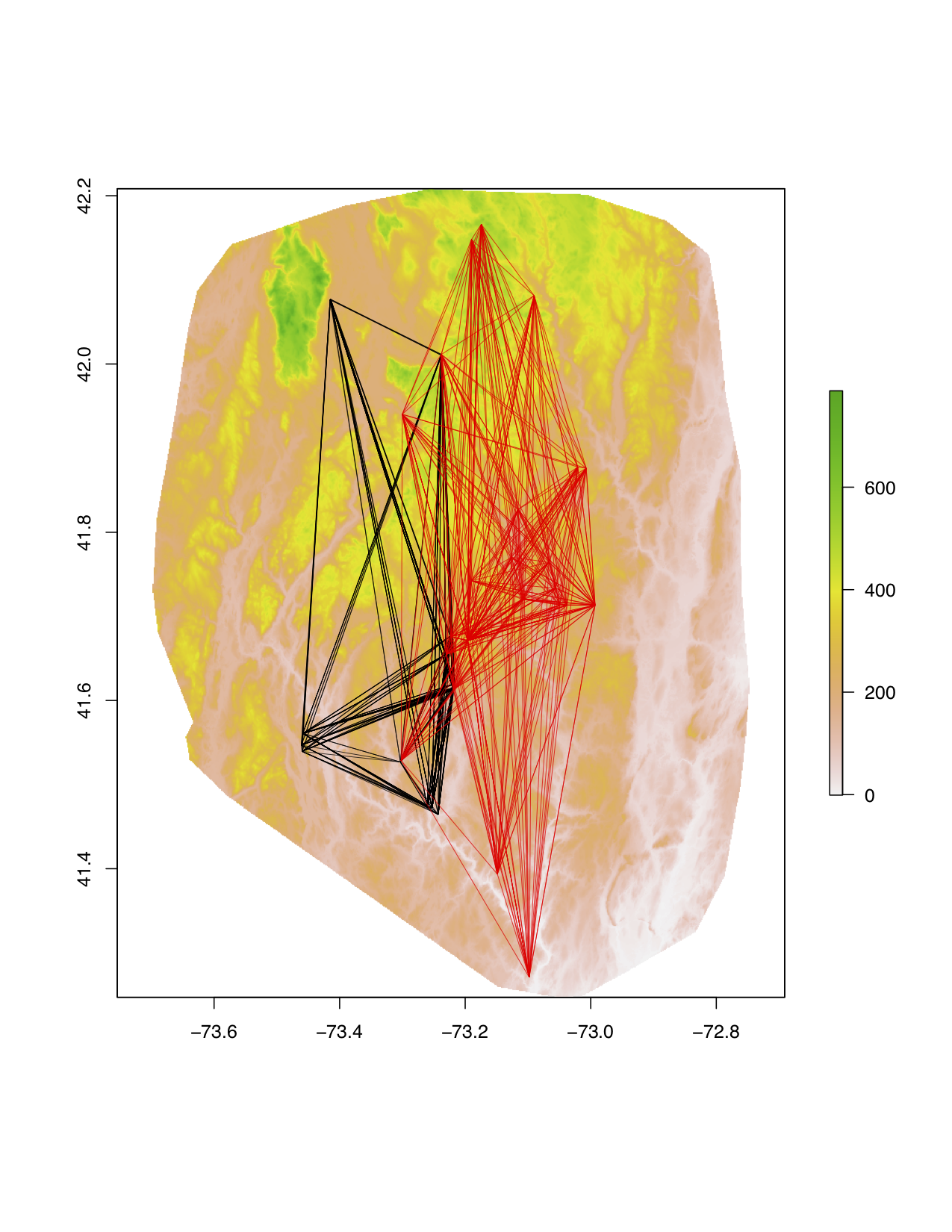


Distance to Development Distance to Road


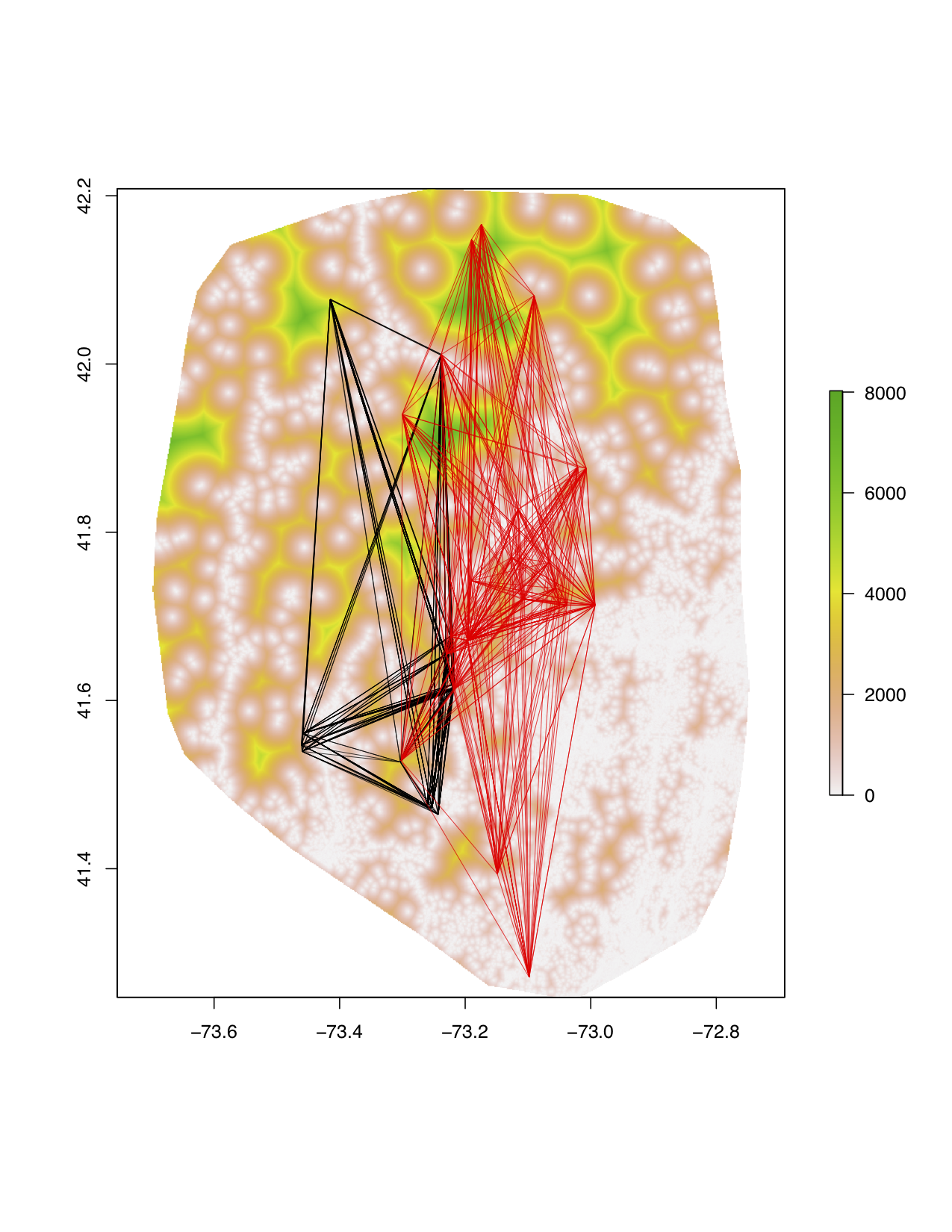

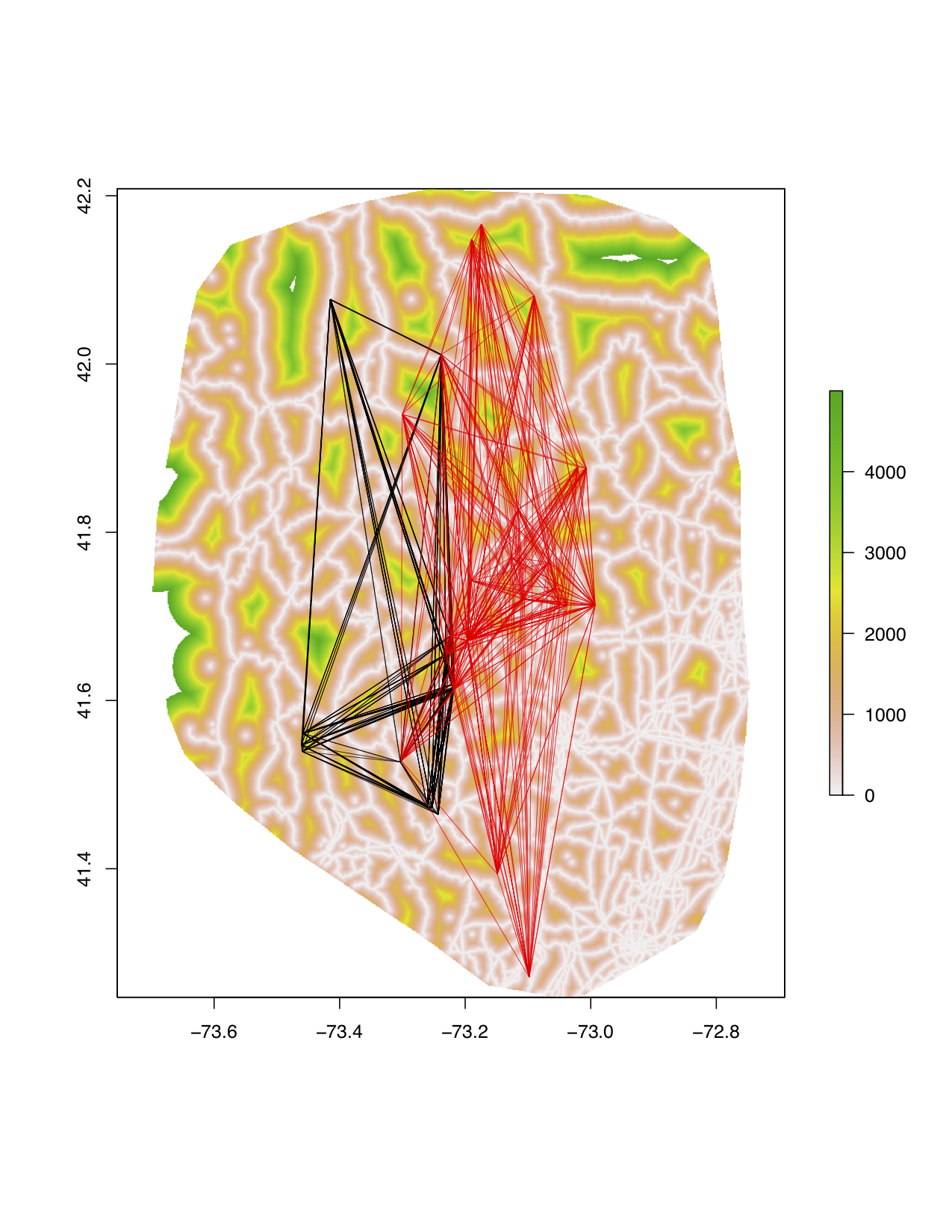


Distance to Shrub Cover Distance to Wetlands


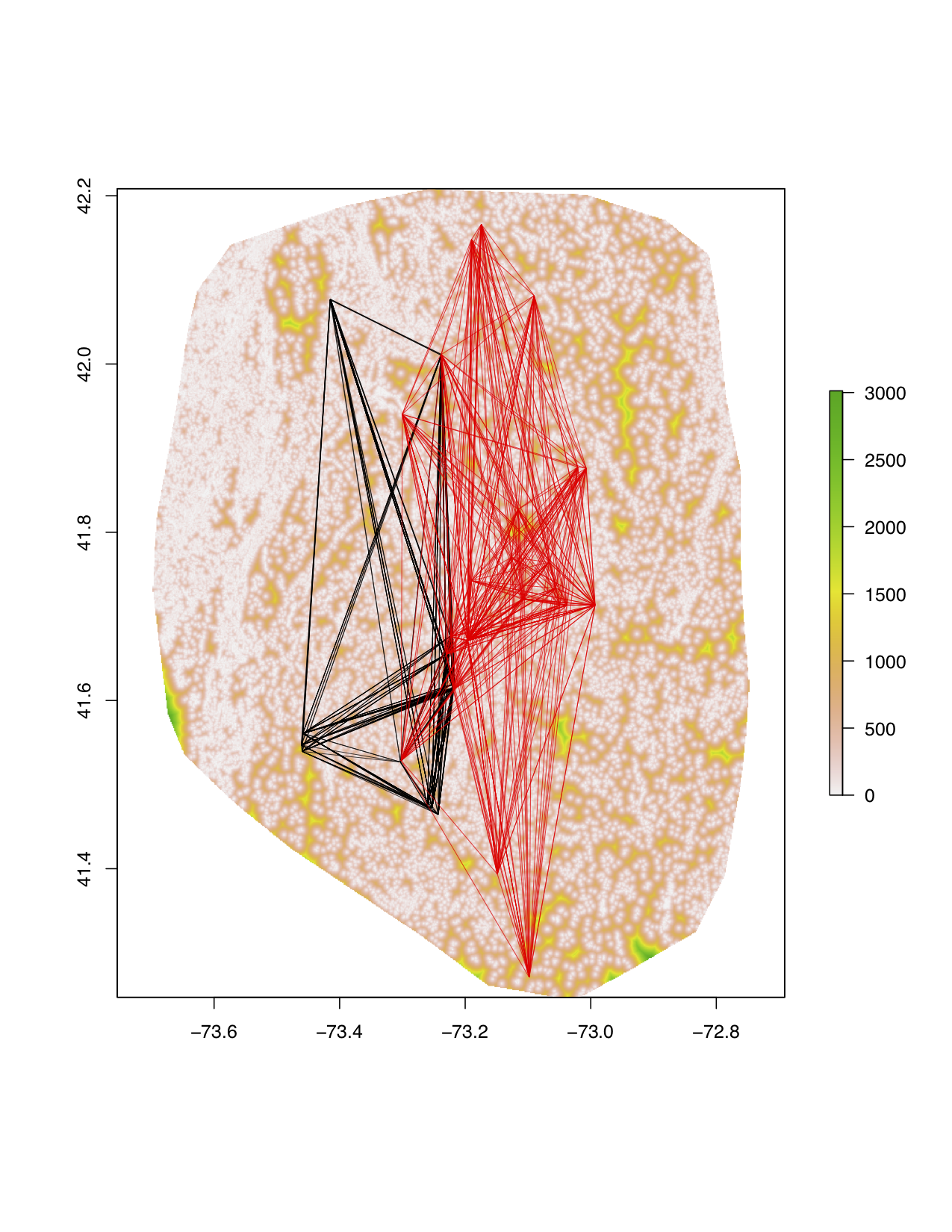

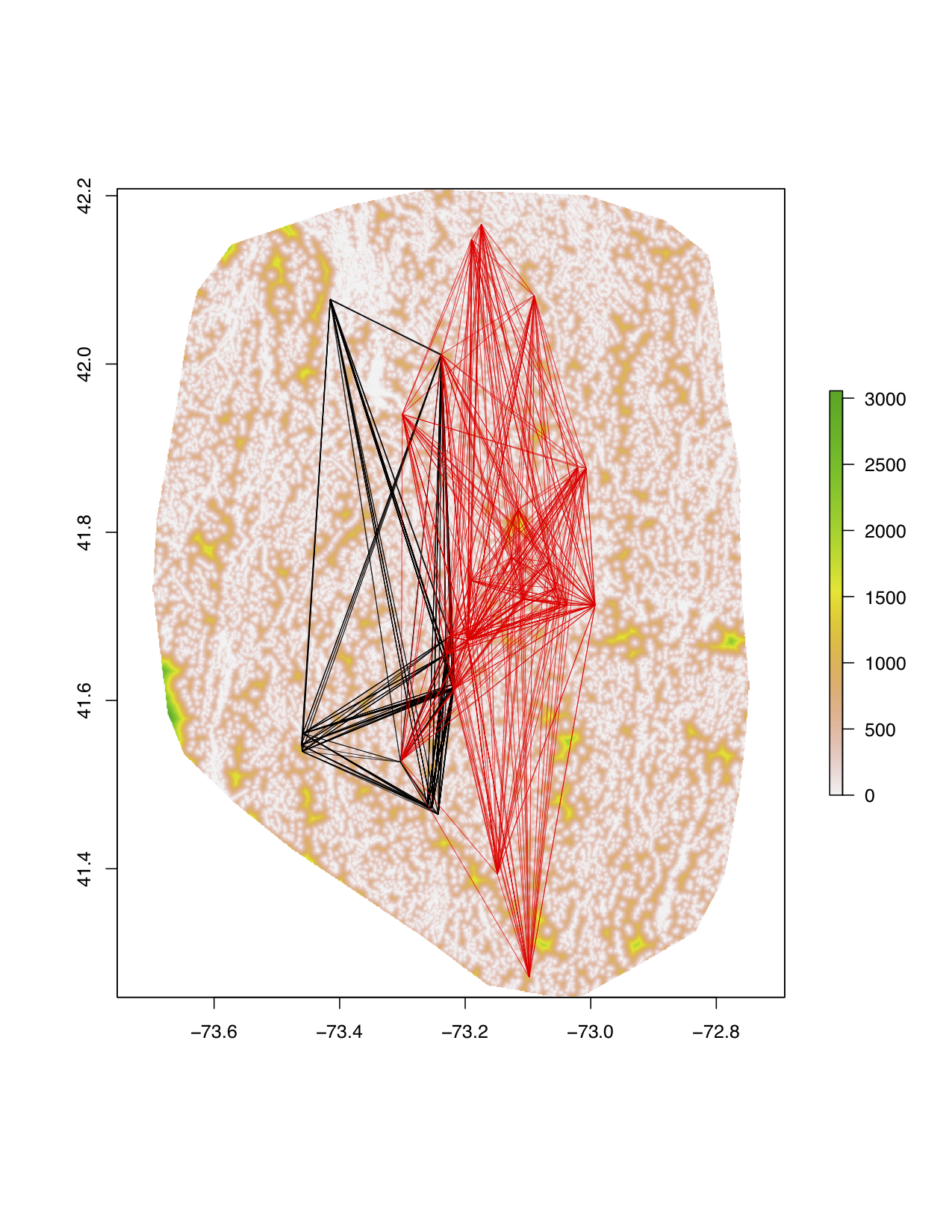


Forest Height


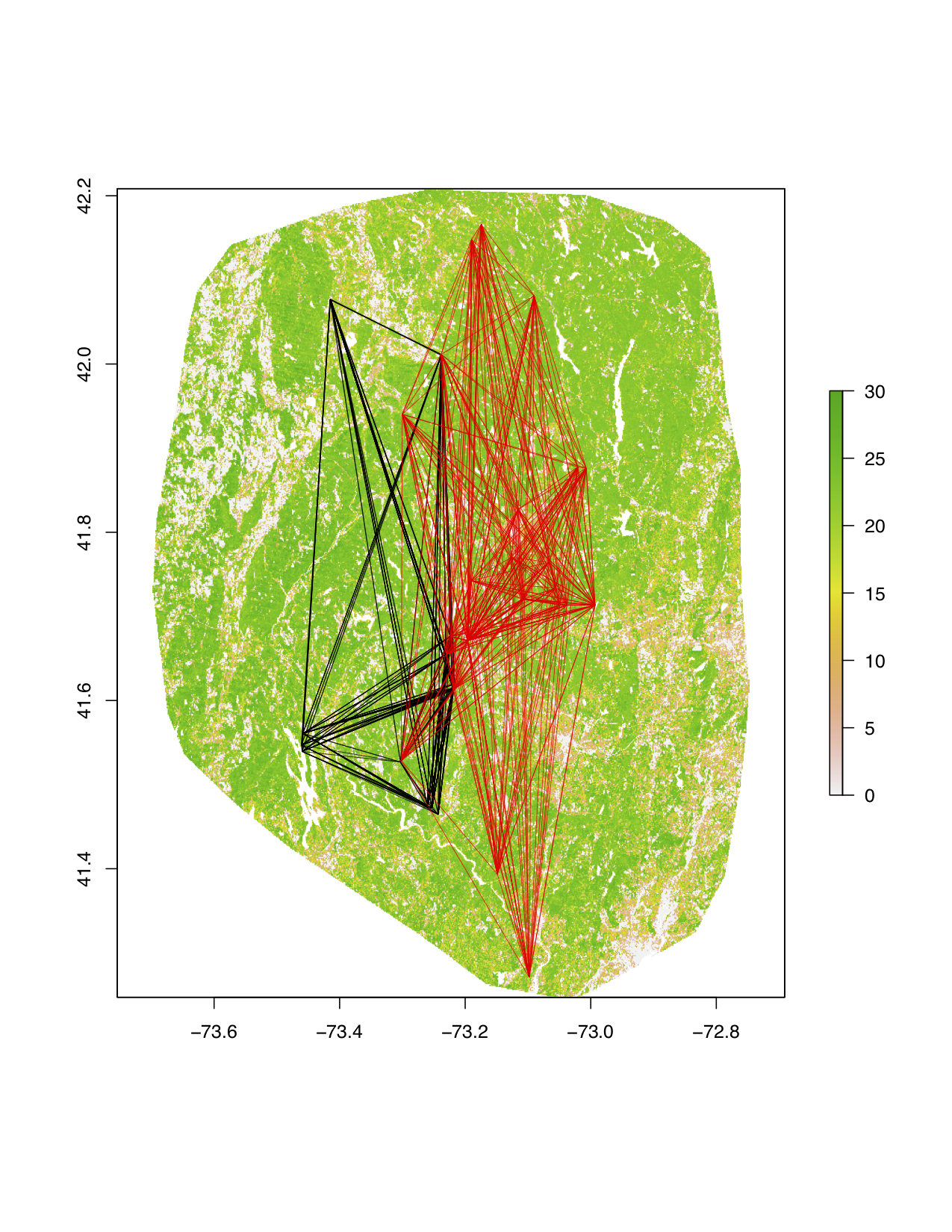


1. East

Aspect DEM


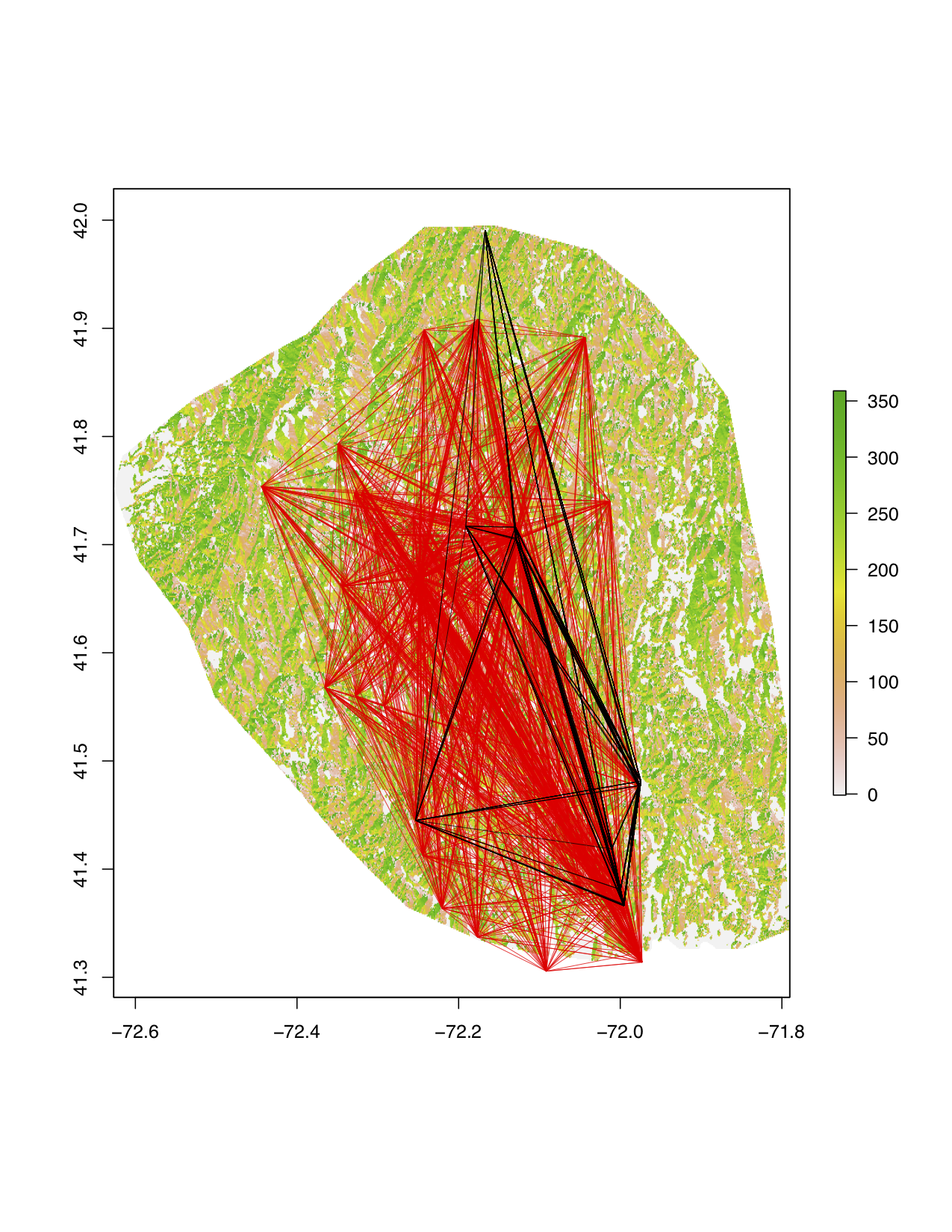

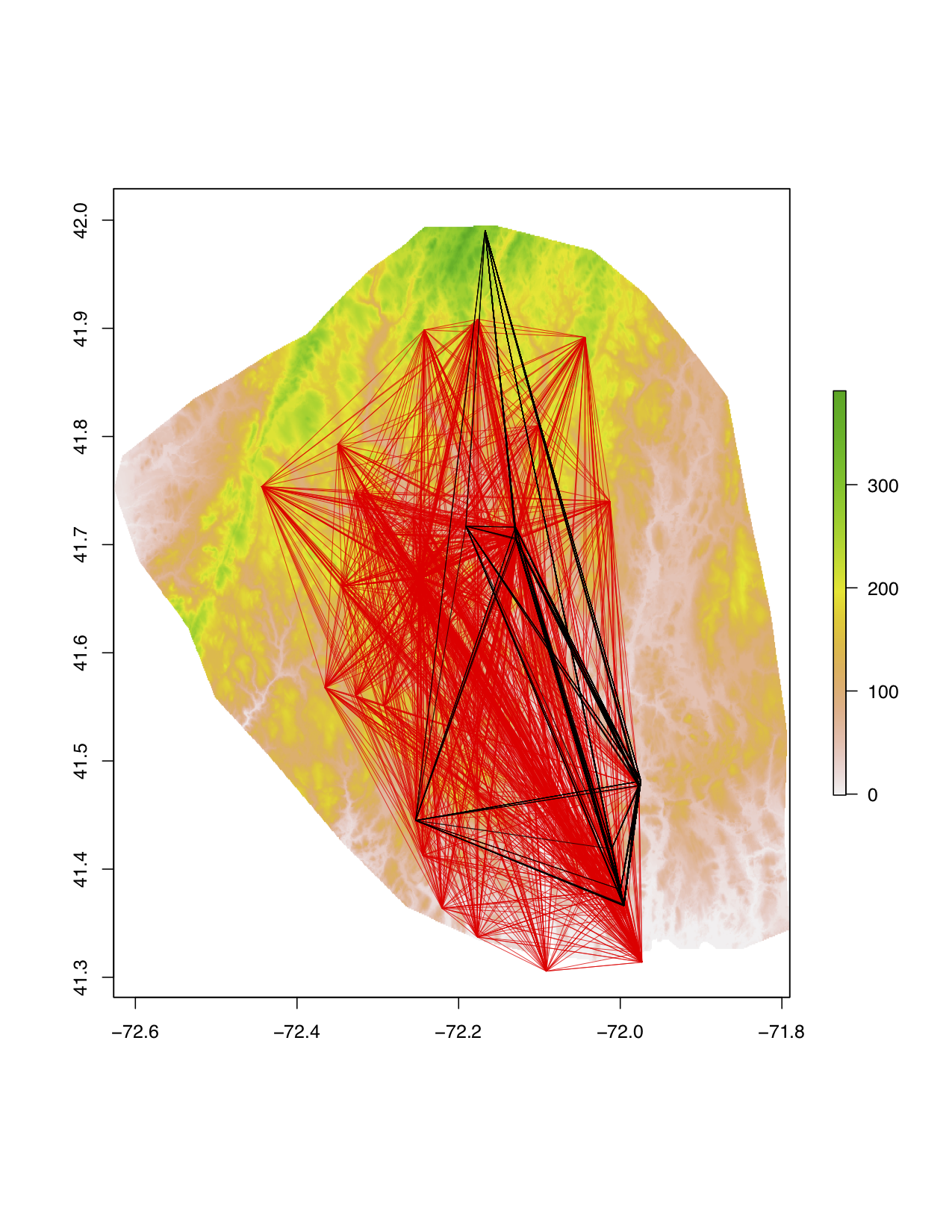


Distance to Development Distance to Road


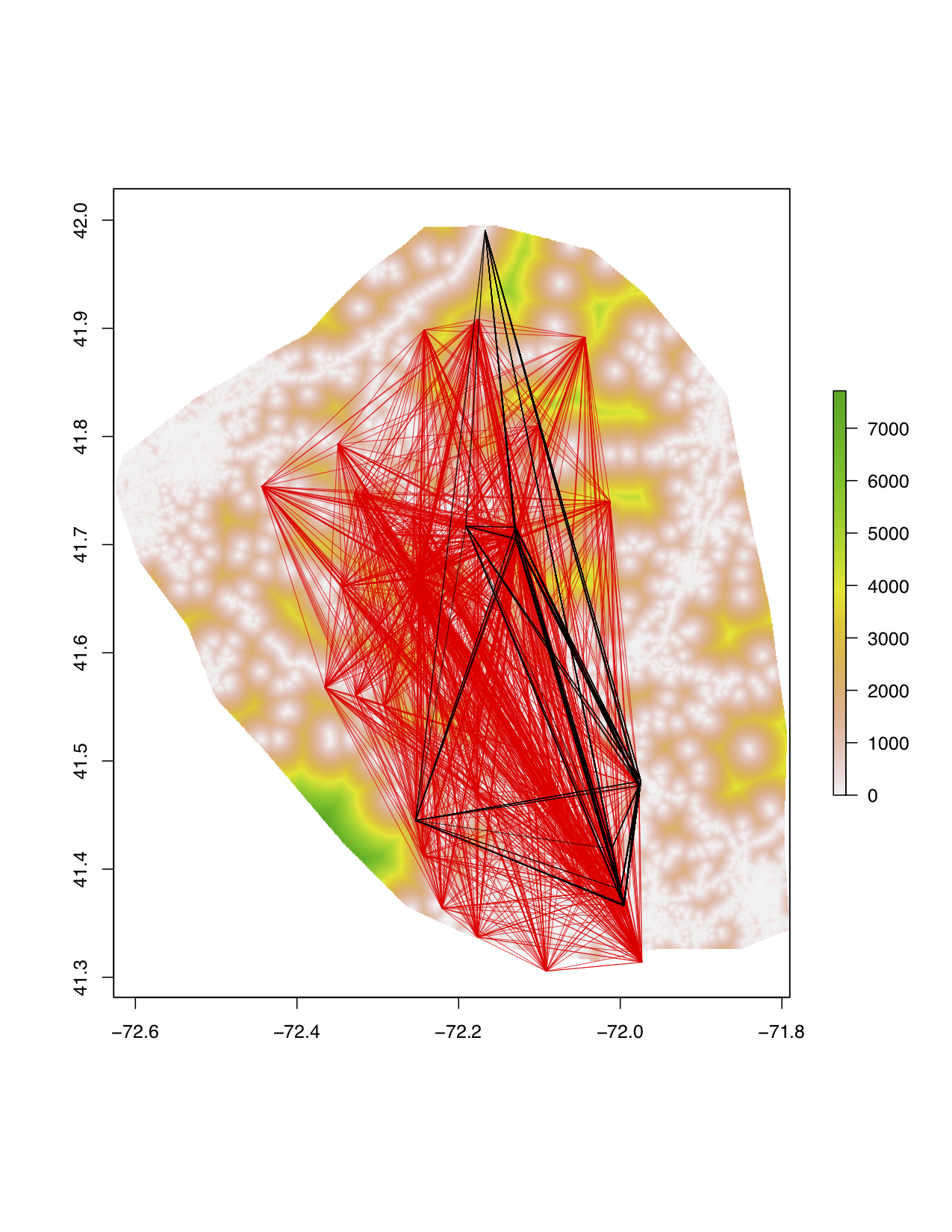

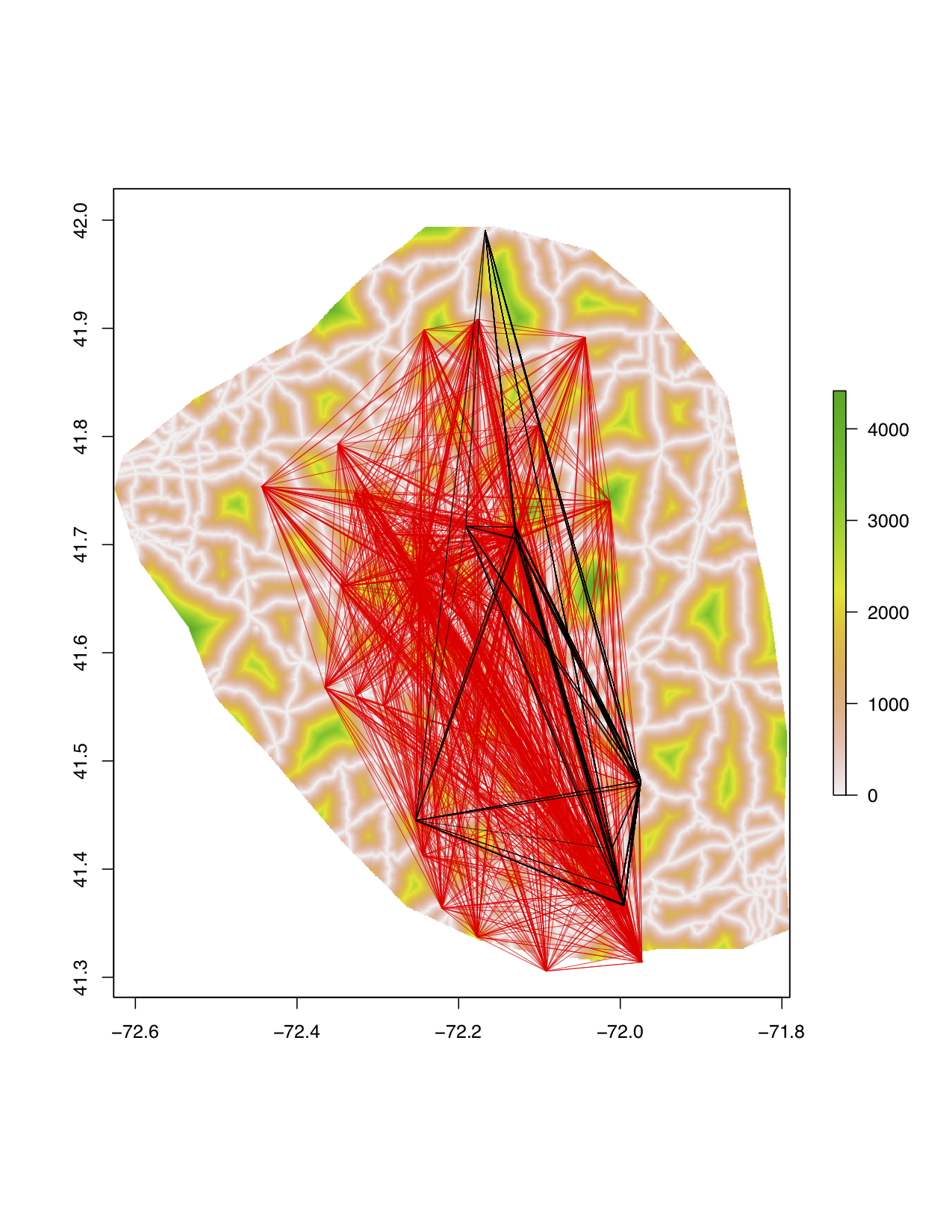


Distance to Shrub Cover Distance to Wetlands


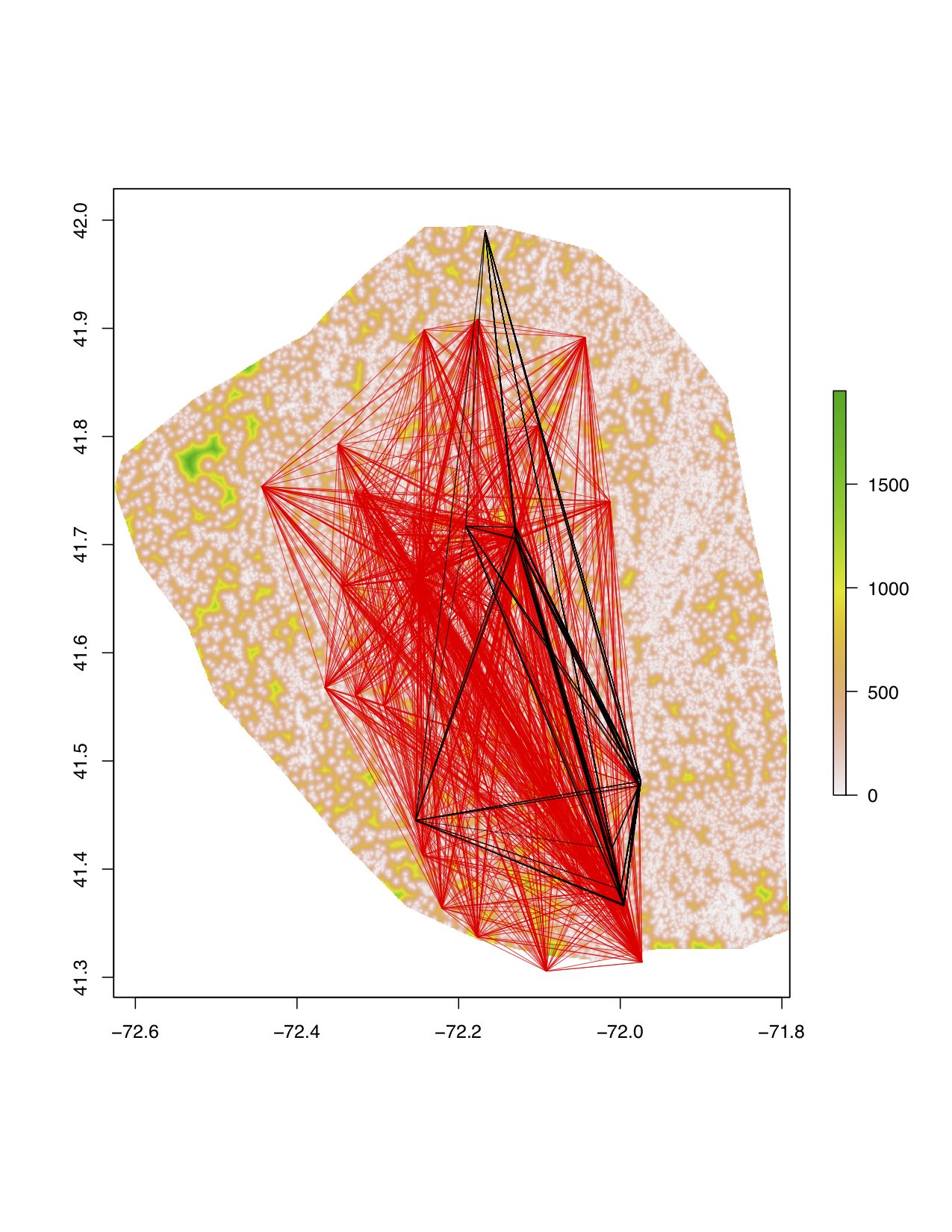

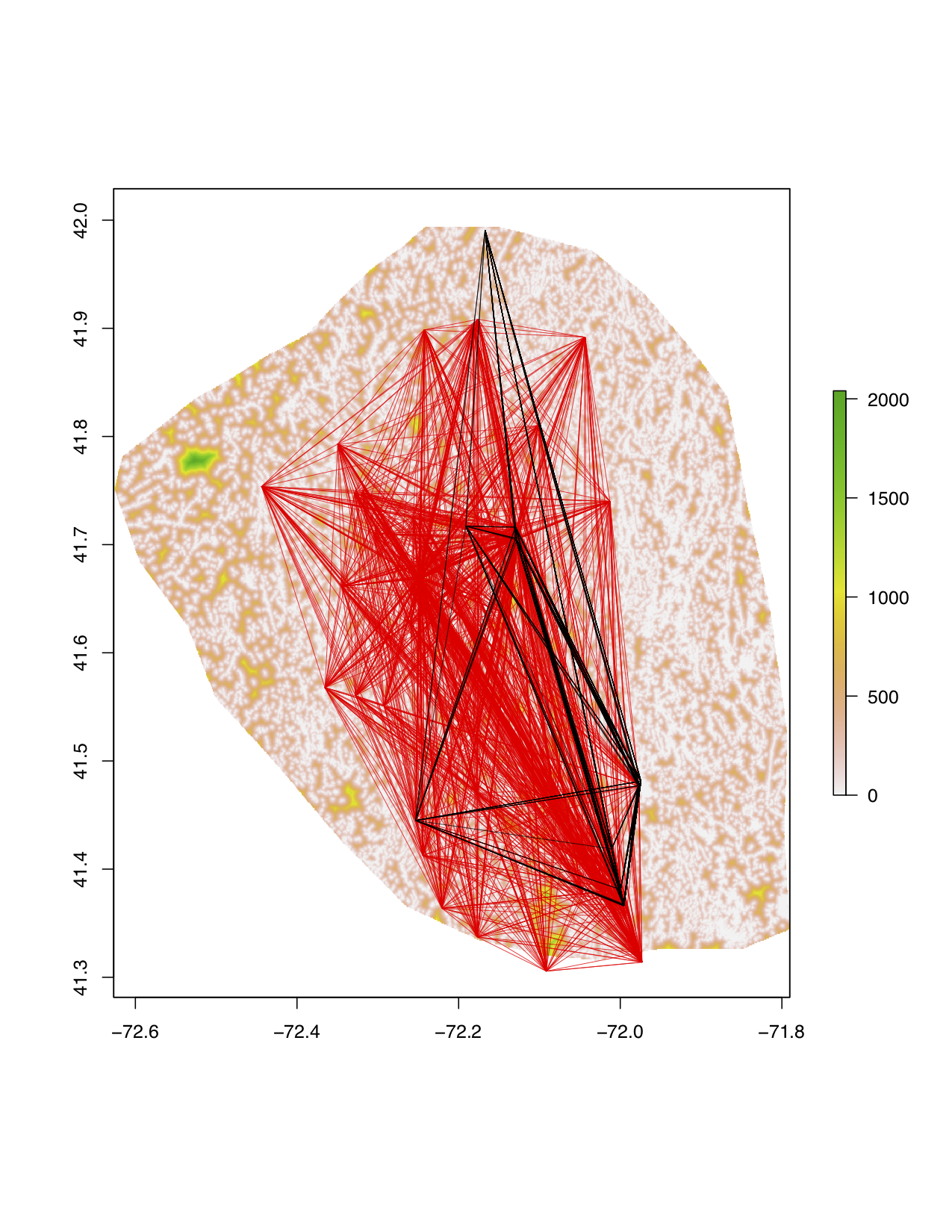


Forest Height


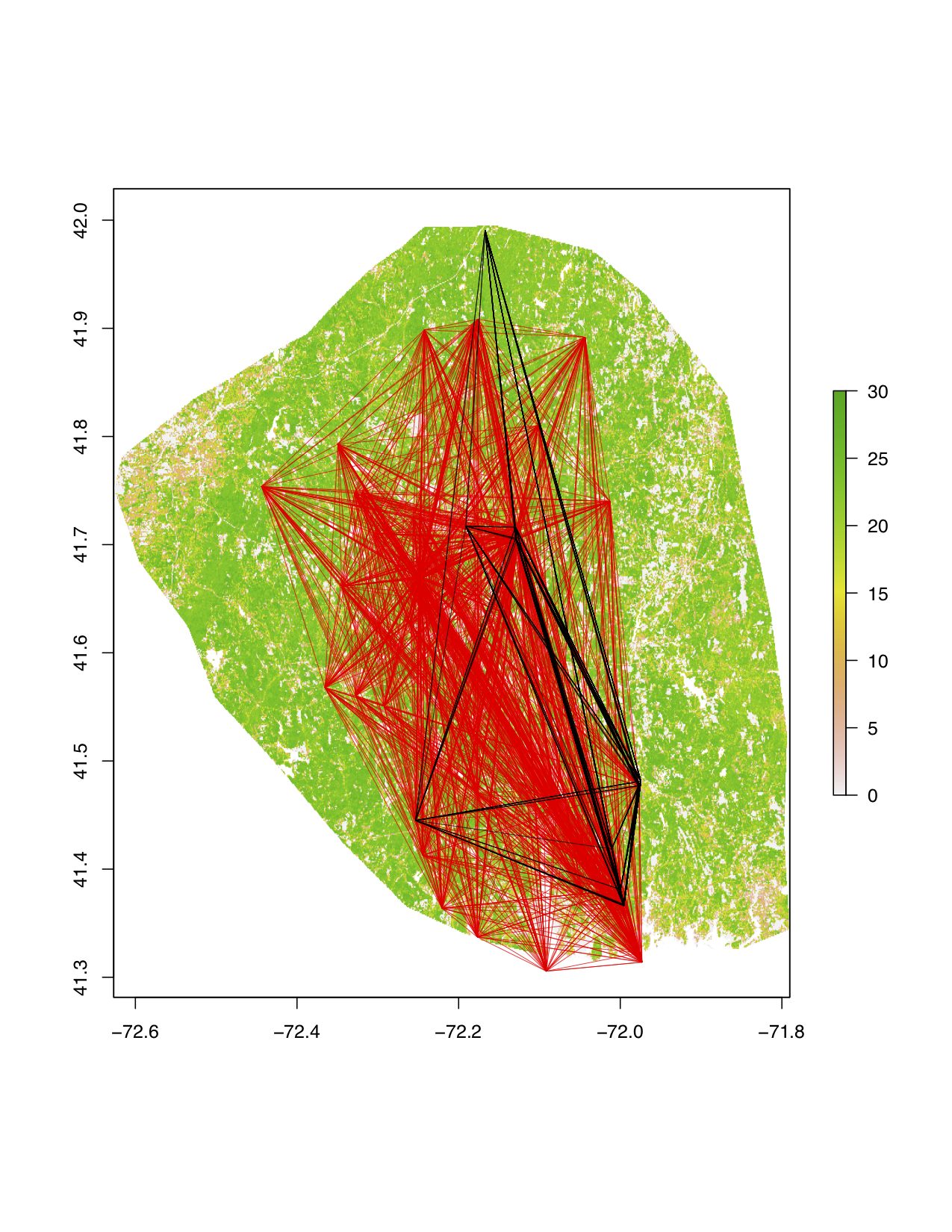


# Data Availability Statement

The datasets generated and analyzed for this study can be found in the NCBI’s SRA database under BioProject accession number PRJNA736852 [https://www.ncbi.nlm.nih.gov/sra].
